# The effect of physical activity on health outcomes in people with moderate-to-severe traumatic brain injury: a rapid systematic review with meta-analysis

**DOI:** 10.1186/s12889-022-14935-7

**Published:** 2023-01-09

**Authors:** Liam Johnson, Gavin Williams, Catherine Sherrington, Kavya Pilli, Sakina Chagpar, Aylish Auchettl, Jack Beard, Renee Gill, Gabrielle Vassallo, Nick Rushworth, Sean Tweedy, Grahame Simpson, Adam Scheinberg, Kelly Clanchy, Anne Tiedemann, Leanne Hassett

**Affiliations:** 1 School of Health Sciences, Faculty of Medicine, Dentistry and Health Sciences, University of Melbourne, Melbourne, Australia; 2 School of Behavioural and Health Sciences, Faculty of Health Sciences, Australian Catholic University, Melbourne, Australia; 3 Physiotherapy Department, Epworth HealthCare, Melbourne, Australia; 4 Sydney School of Public Health, Faculty of Medicine and Health, The University of Sydney, Sydney, Australia; 5 Institute for Musculoskeletal Health, The University of Sydney and Sydney Local Health District, Sydney, Australia; 6 Liverpool Brain Injury Rehabilitation Unit, South Western Sydney Local Health District, Sydney, Australia; 7 Consumer representative, Sydney, Australia; 8 Brain Injury Australia, Sydney, Australia; 9 School of Human Movement and Nutrition Sciences, Faculty of Health and Behavioural Sciences, University of Queensland, Brisbane, Australia; 10 John Walsh Centre for Rehabilitation Research, Kolling Institute, Northern Sydney Local Health District, The University of Sydney and Northern Sydney Local Health District, Sydney, Australia; 11 Murdoch Children’s Research Institute, Melbourne, Australia; 12 School of Medicine, Faculty of Medicine, Dentistry and Health Sciences, University of Melbourne, Melbourne, Australia; 13 School of Health Sciences and Social Work, Griffith Health, Griffith University, Gold Coast, Australia; 14 Menzies Health Institute of Queensland, Griffith University, Gold Coast, Australia; 15 Sydney School of Health Sciences, Faculty of Medicine and Health, The University of Sydney, Sydney, Australia; 16 The University of Sydney, Susan Wakil Health Building, D19 Western Ave, Camperdown, NSW 2006 Australia

**Keywords:** Traumatic brain injury, Disability, Physical activity, Rapid review, Meta-analysis

## Abstract

**Background:**

In 2020, the World Health Organization (WHO) released the first global physical activity and sedentary behaviour guidelines for children and adults living with disability. The evidence informing the guidelines though is not specific to people living with traumatic brain injury (TBI), but rather comes from other disabling conditions such as Parkinson’s disease, and stroke. There remains a clear lack of direct evidence of the effects of physical activity for people living with TBI. The objective of this rapid review was to identify direct evidence of the effect of physical activity on health outcomes in people with moderate-to-severe TBI to inform adaptation of the WHO physical activity guidelines into clinical practice guidelines.

**Methods:**

We conducted a rapid systematic review with meta-analysis of randomised controlled trials, including people of any age with moderate-to-severe TBI, investigating physical activity interventions compared to either usual care, a physical activity intervention with different parameters, or a non-physical activity intervention. Four databases (CENTRAL, SPORTDiscus, PEDro, Ovid MEDLINE) were searched from inception to October 8, 2021. The primary outcomes were physical function, cognition, and quality of life.

**Results:**

Twenty-three studies were included incorporating 812 participants (36% females, majority working-age adults, time post-TBI in studies ranged from 56 days (median) to 16.6 years (mean)). A range of physical activity interventions were evaluated in rehabilitation (*n* = 12 studies), community (*n* = 8) and home (*n* = 3) settings. We pooled data from the end of the intervention for eight outcomes. Participation in a virtual reality physical activity intervention improved mobility, assessed by the Community Balance and Mobility Scale (range 0 to 96; higher score indicates better mobility) more than standard balance training (two studies, 80 participants, Mean Difference = 2.78, 95% CI 1.40 to 4.16; low certainty evidence). There was uncertainty of effect for the remaining outcomes, limited by small sample sizes, diverse comparators and a wide range of outcome measures.

**Conclusion:**

This review consolidates the current evidence base for the prescription of physical activity for people with moderate-to-severe TBI. There remains a pressing need for further rigorous research in order to develop practice guidelines to support clinical decision-making when prescribing physical activity in this population.

**Supplementary Information:**

The online version contains supplementary material available at 10.1186/s12889-022-14935-7.

## Background

Traumatic brain injury (TBI) is a leading cause of death and long-term disability across all ages [1, 2], and can occur at any time across the lifespan [3]. TBI can have both acute and chronic effects, leading to reduced independence and poorer quality of life [4]. People living with TBI exhibit cardiorespiratory dysfunction and exercise intolerance, putting them at high risk of developing chronic health conditions, such as cardiovascular disease [5, 6].

Physical activity can reduce the risk of chronic health conditions for people living with disability and improve overall mood, cognition, and quality of life [7, 8]. Except people living with TBI are typically inactive [9–12] due to injury-related physical and psychosocial outcomes [13, 14], and environmental/accessibility barriers to participation [15]. Those who are most profoundly inactive account for a disproportionately high percentage of the deaths [16] and healthcare costs [17]. This is particularly the case for people living with moderate-to-severe TBI, who are predominantly more inactive, and contribute disproportionally more to the healthcare burden than people living with mild TBI [18, 19]. While people with moderate-to-severe TBI tend to be inactive throughout their course of recovery, they show an increased risk of developing chronic disease and mortality at 3.5 years post injury [6]. Strategies which target the most inactive and aim to improve cardiovascular health, physical function, cognition, and quality of life across the continuum of care are urgently required [20].

In 2020 the World Health Organization (WHO) released the first global physical activity and sedentary behaviour guidelines for children and adults living with disability [8]. The evidence used to inform the development of the guidelines is from healthy populations and several clinical populations, including Parkinson’s disease, and stroke. Critically, the guidelines do not include direct evidence of the effects of physical activity for people living with TBI or include studies undertaken as part of rehabilitation. This rapid review aims to address this evidence gap.

A rapid review was chosen as “a form of knowledge synthesis that accelerates the process of conducting a traditional systematic review…to produce evidence for stakeholders in a resource-efficient manner” [21]. The primary objective of this rapid review was to assess the effects of physical activity on physical function, cognition, and quality of life across the lifespan and continuum of care for people living with moderate-to-severe TBI. Secondary objectives were to assess the effects of physical activity on mortality, comorbid conditions, mood, participation and levels of physical activity. Along with other studies planned and underway by our research team, this review will contribute to the adaptation of WHO guidelines into clinical practice guidelines for Australian healthcare services working with children, adolescents, adults, and older adults living with moderate-to-severe TBI.

## Methods

A rapid systematic review was used to perform an accelerated, time-limited review of relevant TBI literature [21]. The Cochrane Rapid Review Methods Group Guidelines [22] were adhered to in performing this review, and reporting followed the Preferred Reporting Items for Systematic Reviews and Meta-Analyses (PRISMA) guidelines (Appendix 1) [23]. This review has been completed in accordance with the study protocol registered in PROSPERO (CRD42021284036) prior to commencement. There were no deviations from the protocol registered on PROSPERO.

### Search strategy

A systematic literature search of four databases (CENTRAL, SPORTDiscus, PEDro and Ovid MEDLINE) was performed to capture appropriate studies from database inception to October 8, 2021 (see Appendix 2 for the full search strategy for all four databases). The search strategy was developed by authors LH and KP and reviewed by a University of Sydney Health Sciences librarian. The MEDLINE search strategy was independently peer reviewed by a colleague with expertise in TBI and conducting systematic reviews. Reference lists of relevant systematic reviews, trial registries and protocols, and included full-text articles, were hand searched to ensure no studies were overlooked. Non-English language studies, non-human studies, and conference abstracts were excluded.

### Study selection criteria

#### Study type

Randomised controlled trials (RCTs) testing the effects of physical activity on health outcomes in people with moderate-to-severe TBI were targeted for inclusion. Cross-over RCTs were also included, but only data reported from the first phase of the cross-over trial.

#### Population

Trials involving people of any age with moderate-to-severe TBI at any time post-injury, and only studies where at least 50% of participants had a moderate-to-severe TBI (or for whom separate data for participants with TBI were available) were included. Where not specifically indicated in the article, authors were contacted for further details on the injury severity of the participants included in their study. If no response was forthcoming, the study was excluded from the review. Moderate injury was defined as post-traumatic amnesia (PTA) [24] between one to seven days and/or an altered level of consciousness (Glasgow Coma Scale {GCS} [25] score 9 to 12) or loss of consciousness between 30 min and 24 h post-trauma. Severe injury was defined as PTA duration longer than seven days, or a period of coma with GCS score of eight or less or a loss of consciousness greater than 24 h [26].

#### Intervention

We considered a physical activity intervention to be any intervention that would contribute to the participant meeting the WHO physical activity guidelines. This includes structured exercise (i.e., aerobic; strength; gait/balance/functional; or multicomponent training), sport and physical recreation, or any intervention that aimed to promote overall physical activity (e.g., health coaching, pedometer programs). The physical activity may be delivered as a standalone intervention or as part of a rehabilitation package and may be supervised or self-led. The intervention may be implemented at any point along the continuum of care and in any setting. The physical activity intervention had to be of a minimum two-weeks duration and could be prescribed alone or as a component of an intervention, where physical activity is > 50% of the intervention. In instances where physical activity was ‘assisted’ (i.e., robotics, body-weight support), studies were included if the intervention required the participant to produce at least 50% voluntary/unassisted activity.

#### Comparator

To be eligible, studies had to compare one or more groups that completed a physical activity intervention to either (i) usual care, (ii) a physical activity intervention with different parameters, such as dose, setting, or supervision, (iii) a non-physical activity intervention, or (iv) no intervention.

#### Outcome measures

We included any relevant health-related outcomes under the following outcome domains: physical function, cognition, and quality of life (primary objectives); physical activity, participation, comorbidities and mortality, and psychological function (secondary objectives). We also assessed the incidence of adverse events in the included studies. The outcomes used in this review are aligned with those evaluated in the development of the WHO physical activity and sedentary behaviour guidelines for people living with disability [8], as well as additional outcomes considered by the authors (including people with lived experience) of importance for people living with moderate-to-severe TBI.

### Data management and selection procedure

Articles were initially imported into Endnote before duplicates were removed and the remaining records were imported into a web-based data management platform (Covidence 2020 v1517, Melbourne, Australia) for screening. Using the eligibility criteria, a team of six reviewers screened the titles and abstracts of the imported studies. Initially, the same 50 records were screened by the entire screening team to calibrate and test the review form. Then, two reviewers independently screened all remaining records, with conflict resolution completed by a third reviewer (LJ). The same team of reviewers completed the full text screening. Each full text record was screened by two reviewers independently, with studies excluded based on the predetermined exclusion criteria. Conflict resolution was completed by a third reviewer (LJ).

### Data extraction

Data extraction was completed by a single reviewer from the review team using a self-developed, customised data extraction template in a Microsoft Excel spreadsheet. A second reviewer (LJ) checked the extracted data for correctness and completeness. The data extraction form was developed and piloted on two studies initially by two reviewers (SC and LJ). Data extraction included information on study design, setting, location, sample size, sample characteristics, intervention components, outcome measures, and key findings. In instances of mixed study populations (i.e., mild, moderate and severe TBI, TBI and other acquired brain injuries), where possible, only moderate-to-severe TBI data were extracted. If this was not possible, group data was used in the synthesis and analysis. Where multiple measures were used in a single study to assess the same, or similar, construct, the authors chose the measure they believed most appropriately measured the construct given their experience in the field and knowledge of the literature.

### Quality appraisal

Study quality was assessed using the Physiotherapy Evidence Database (PEDro) scale [27]. Quality assessments of RCTs included in the review were obtained from the PEDro database (see http://www.pedro.org.au). Every study was assigned a score (0–10), with a lower rating indicating a higher risk of bias, while a score of ≥ 7 represents a study of moderate to high quality [28]. No studies were excluded based on the quality appraisal.

### Data synthesis

We synthesised the details of the population, intervention, comparison and measured outcomes in Tables 1 and 2. For outcomes measured on the same scale, we calculated the mean difference (MD) (difference in means) and 95% confidence intervals (CI) using a random-effects model. Where outcomes were measured using different assessments/measures, we calculated the standardised mean difference (SMD) (Hedges’ g) and 95% CI using a random-effects model to pool estimates. Mean and standard deviations were used where reported in the included studies. Where median and interquartile range (IQR) were reported, the mean and SD were calculated as per the quantile estimation method described by McGrath et al. (2020) [29]. Where change scores were reported, these were pooled with end of intervention and/or end of follow-up scores for analysis but are presented for these studies as separate subgroups [30]. Where data were reported in figures only in the included studies, we used WebPlotDigitizer [31] to extract numerical data. Effect sizes were categorised as small (0.1 to 0.4), medium (0.5 to 0.7) or large (0.8 or greater) [32]. Heterogeneity was determined by visual inspection of the forest plots and with consideration of the I^2^ test. Interpretations of the effect of the intervention were based on visual inspection of the forest plots (i.e., similarity of point estimates, overlapping of confidence intervals), the tests of significance and the confidence intervals presented in the forest plots generated. We did not test for publication bias due to the small number of studies included in the meta-analysis. Overall grading of the evidence related to each primary outcome that was synthesised in meta-analysis was determined using the GRADE approach [33]. For outcomes not included in the meta-analysis, we calculated the MD and 95% CIs for each outcome at end of intervention and end of follow-up where indicated.

**Table 1:** Participant and study characteristics

Reference	Country	Setting	Sample Size	Female Sex (*n*=)/%)	Age (years) Mean ± SD	Injury severity (*n*=) Moderate/ Severe)	Time post-Injury Mean ± SD (months)^+^	PEDro Quality Assessment for RCTs (total)	Control Group comparison
Bateman et al. 2001 [34]	UK	Inpatient Rehab	I: 24 C: 23	I: 6 (25%) C: 3 (13%)	I: 35 ± 14 C: 36 ± 13	I: 0/14 C: 0/12	I: 5.1 ± 2.9^a^ C: 5.0 ± 2.2	7	Non-PA intervention (relaxation)
Bellon et al. 2015 [35] & Kolakowsky-Hayner et al. 2017 [36]^b^	USA	Home-based	I: 29 C: 40	28 (41%)	44 ± 16	10/35	100.5 ± 119.9	6^c^	Non-PA intervention (nutrition coaching)
Blake et al. 2009 [37]	UK	Community-based	I: 10 C: 10	I: 1 (10%) C: 4 (40%)	I: 44 ± 10 C: 46 ± 11	I: 4/3 C: 4/2	I: 196.8 ± 108.0 C: 178.8 ± 163.2	6	Non-PA intervention (social & leisure activities)
Brenner et al. 2012 [38]	USA	Community-based	I: 37 C: 37	I: 8 (22%) C: 5 (14%)	I: 44 ± 16 C: 44 ± 15	I: NR C: NR	I: 140.4 ± 165.6 C: 150.0 ± 165.6	4	No intervention (wait-list)
Brown et al. 2005 [39]^d^	USA	Inpatient Rehab	I: 10 C: 9	I: 3 (30%) C: 3 (33%)	I: 38 ± 12 C: 42 ± 8	I: 0/10 C: 0/9	I: 181.2 ± 78.0 C: 199.2 ± 199.2	5	PA intervention (overground gait training)
Canning et al. 2003 [40]^e^	Australia	Inpatient Rehab	I: 13 C: 11	I: 2 (16%) C: 4 (40%)	I: 25 ± 11 C: 26 ± 10	I: 0/13 C: 0/11	I: 2.5 ± 1.5 C: 2.8 ± 0.8	7	No additional intervention (usual rehab)
Curcio et al. 2020 [41]^e^	Italy	Inpatient Rehab	I: 11 C: 11	I: 6 (60%) C: 5 (50%)	I: 37 ± 15 C: 43 ± 14	I: 0/11 C: 0/11	I: 5.8 ± 2.6 C: 4.8 ± 2.7	6	PA intervention (balance training)
Cuthbert et al. 2014 [42]	USA	Inpatient Rehab	I: 10 C: 10	I: 3 (30%) C: 4 (40%)	I: 32 (23–56)^f^ C: 31 (19–64)	I: NR C: NR	I: 1.8 (0.9–2.8)^f^ C: 3.1 (0.8-4.0)	6	PA intervention (balance training)
Driver et al. 2004 [43]	USA	Outpatient Rehab	I: 8 C: 8	I: 4 (50%) C: 4 (50%)	I: 39 ± 5 C:38 ± 4	I: 0/8 C: 0/8	I: 40.8 ± 17.1 C: 36.6 ± 14.1	3	Non-PA intervention (vocational rehab)
Driver et al. 2006 [44]	USA	Outpatient Rehab	I: 9 C: 9	I: 4 (44%) C: 4 (44%)	I: 38 ± 4 C: 35 ± 4	I: 0/9 C: 0/9	I: 40.3 ± 14.7 C: 41.2 ± 14.2	4	Non-PA intervention (vocational rehab)
Driver et al. 2009 [45]	USA	Outpatient Rehab	I: 8 C: 8	I: 3 (38%) C: 4 (50%)	I: 39 ± 2 C: 38 ± 2	I: 8/0 C: 8/0	I: 40.8 ± 14.7 C: 36.2 ± 14.2	4	Non-PA intervention (vocational rehab)
Esquenazi et al. 2013 [46]	USA	Outpatient Rehab	I: 8 C: 8	I: 5 (62%) C: 4 (50%)	I: 37 ± 11 C: 42 ± 17	NR	I: 140.3 ± 71.6 C: 150.4 ± 111.6	4	PA intervention (Manual-Assisted Partial BWSTT)
Freivogel et al. 2009 [47]^g^	Germany	Inpatient Rehab	I: 8 C: 8	I: 3 (38%) C: 2 (25%)	I: 22 ± 6 C: 26 ± 6	I: 0/6 C: 0/6	I: 16.0 ± 15.0 C: 56.0 ± 69.0	8	PA intervention (Partial BWSTT or overground walking)
Gemmell et al. 2006 [48]	New Zealand	Community-based	I: 9 C: 9	All: 9	All: F, 40 ± 12 M, 51 ± 9	I: NR C: NR	All: 104.4	5	No intervention (wait-list)
Hassett et al. 2009 [49] & Hassett et al. 2011 [50]	Australia	Community-based	I: 32 C: 30	I: 5 (15%) C: 4 (12%)	I: 35 ± 15 C: 33 ± 12	I: 0/32 C: 0/30	I: 2.6 (1.8-4.0) C: 2.3 (1.5–3.4)^h^	8	PA intervention (home-based exercise)
Hassett et al. 2012 [51]	Australia	Inpatient & Outpatient Rehab	I: 20 C: 20	I: 6 (30%) C: 7 (35%)	I: 39 ± 17 C: 29 ± 11	I: 0/20 C: 0/20	I: 3.7 (2.0-4.9) C: 3.1 (2.1–5.6)^h^	7	PA intervention (Circuit class, no HR feedback)
Katz-Leurer et al. 2009 [52]^i^	Israel	Home-based	I: 10 C: 10	I: 3 (30%) C: 3 (30%)	I: 8 ± 4 C: 9 ± 3	I: 0/5 C: 0/5	I: NR C: NR	7	No intervention
Kleffelgaard et al. 2019 [53]^j^	Norway	Outpatient Rehab	I: 33 C: 32	I: 23 (70%) C: 22 (71%)	I: 38 ± 12 C: 41 ± 14	I: NR C: NR	I: 3.9 ± 2.2 C: 3.4 ± 1.9	8	No additional intervention (usual rehab)
McMillan et al. 2002 [54]^k^	UK	Community-based	I: 47 C: 48	I: 8 (21%) C: 12 (25%)	I: 31 ± 13 C: 36 ± 13	I: NR C: NR	I: NR C: NR	6	No intervention
Särkämö et al. 2021 [55]^b^	Finland	Outpatient rehab	I: 6 C: 5	I: 3 (50%) C: 1 (20%	I: 36 ± 6 C: 35 ± 14	I: 0/6 C: 0/5	I: 110.4 ± 30.0 C: 69.6 ± 37.2	7	No intervention (wait-list)
Straudi et al. 2017 [56]^l^	Italy	Inpatient & Outpatient Rehab	I: 11 C: 10	I: 2 (16%) C: 2 (22%)	I: 30 ± 16 C: 37 ± 10	I: NR C: NR	I: 24.0 ± 72.0 C: 96.0 ± 192.0	7	PA intervention (balance training)
Tefertiller et al. 2019 [57]	USA	Home-based	I: 31 C: 32	I: 8 (26%) C: 16 (50%)	I: 48 ± 12 C: 50 ± 12	I: NR C: NR	I: 99.6 ± 110.4 C: 102.0 ± 87.6	7	PA intervention (home-based balance training)
Wilson et al. 2006 [58]^e^	USA	Inpatient Rehab	I: 20 C: 20	I: 1 (5%) C: 2 (11%)	I: 33 ± 14 C: 26 ± 9	I: NR C: NR	I: 4.0 ± 3.5 C: 2.8 ± 1.8	7	No additional intervention (usual gait rehab)

*SD*, standard deviation, *PEDro* Physiotherapy Evidence Database *RCT* Randomised Controlled Trial, *UK* United Kingdom, *Rehab* Rehabilitation, *I* Intervention, *C* Control, *PA* Physical Activity, *USA* United States of America, *NR* not reported, *BWSTT* Body-Weight Supported Treadmill Training, *F* Female, *M* Male, *HR* Heart Rate

^+^ Where time post-injury was reported in days, weeks or year/s, the mean and SD were converted to months

^a^ In Bateman et al. [34] for the time post-injury, *n* = 23. TBI-specific data from this study is taken from email correspondence with author LH from Cochrane review (Hassett et al., 2017) [59]

^b^ Study employed a cross-over RCT design. The demographic data reported here is from the first phase of the trial only

^c^ The PEDro score reported here is that of the Bellon et al. [35] article. The Kolakowsky-Hayner et al. [36] article has been scored as a 4 according to the PEDro database. We have chosen to report the higher of the two PEDro scores for this study

^d^ The number of moderate/severe TBI participants is not reported in the article [39], but was confirmed via personal correspondence with the lead author

^e^ Demographic data presented excludes participants lost to follow-up

^f^ Median and Range (min-max.)

^g^ In Freivogel et al. [47], of the *n* = 8 participants randomised to each of the experimental and control groups, 6 were diagnosed TBI, 1 was diagnosed stroke, and 1 was diagnosed spinal cord injury in each group. The demographic data provided is based on the total group (i.e., *n* = 8) for both the experimental and control groups

^h^ Median and interquartile range

^i^ In Katz-Leurer et al. [52], of the *n* = 10 participants randomised to each of the experimental and control groups, 5 were diagnosed TBI and 5 were diagnosed cerebral palsy in each group. The demographic data provided is based on the total group (i.e., *n* = 10 TBI  + cerebral palsy) for both the experimental and control group

^j^ In Kleffelgaard et al. [53], *n* = 32 participants were initially randomised to the control group. But one participant did not receive the allocated control due to not wishing to participate. The demographic data provided for the control group is based on *n* = 31. All demographic data is provided for mild-to-moderate severe TBI.

^k^ In McMillan et al. [54], *n* = 145 were initially randomised to three groups, an attentional training group and a physical exercise group, and a no intervention control group. In this review, only the physical exercise experimental group has been included for comparison with the no intervention control group. Nine participants from the physical exercise experimental group failed to complete the treatment. Therefore, the demographic data reported here for the experimental group is *n* = 38

^l^ In Straudi et al. [56], *n* = 11 participants were initially randomised to the experimental group, but one participant randomised to the control group mistakenly received the experimental intervention. Therefore, the demographic data for the experimental group is based on *n* = 12, and for the control group is *n* = 9

## Results

### Search results and overview

**Fig. 1:**
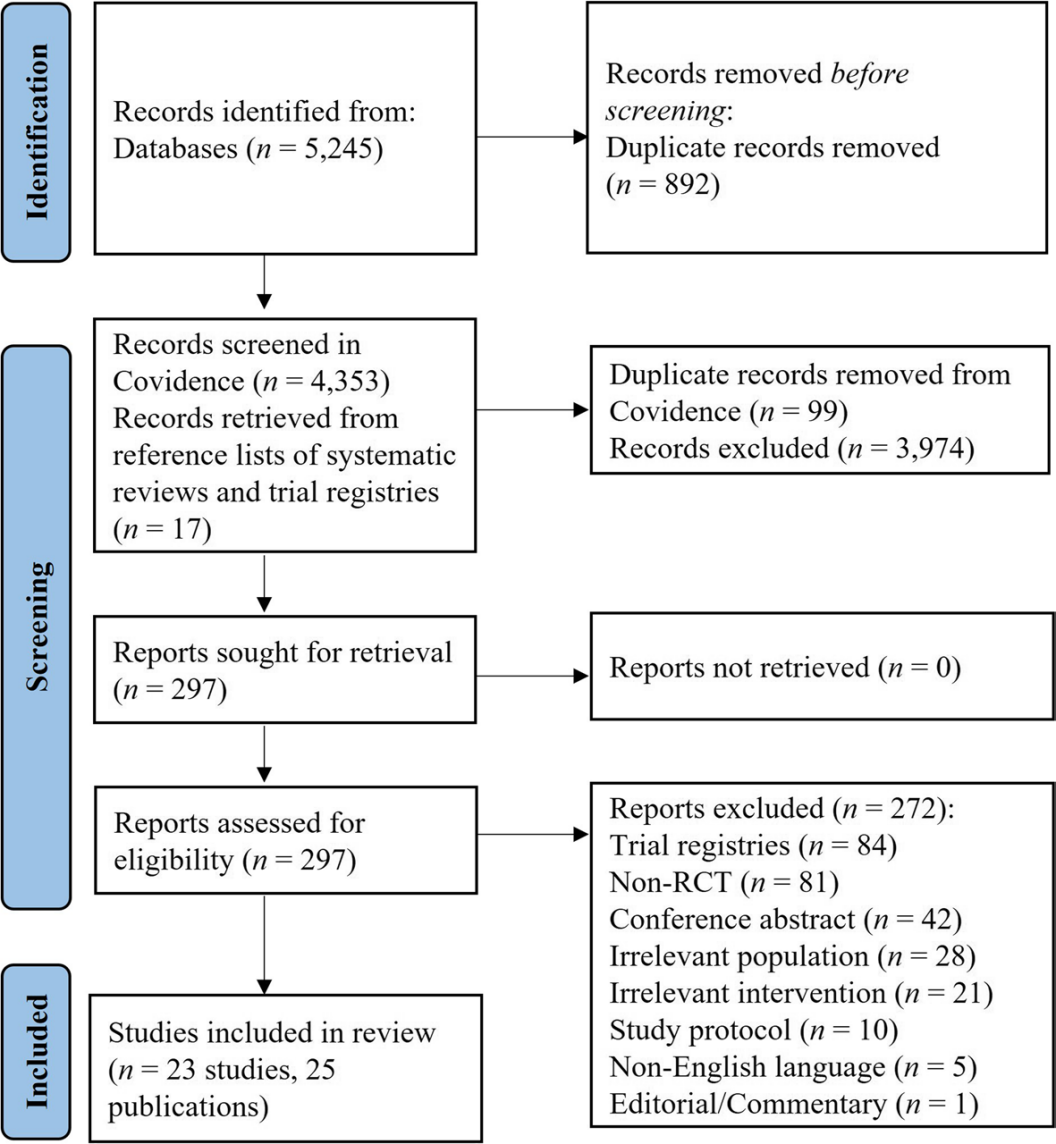
Results of a systematic search process [60]

The literature search yielded a total of 5,245 articles, of which 4,353 were screened for eligibility after duplicates were removed. A total of 4,073 were excluded following title and abstract screening, leaving 297 articles for full text review. Following full-text screening, 272 papers were excluded as they did not satisfy the inclusion criteria of this review. This left 25 articles describing 23 studies. (Flow of records is summarized in Fig. 1).

### Study characteristics

Of the 23 included studies, two employed a cross-over trial design [35, 36, 55], one of which contributed two articles in this review [35, 36]. One study included a secondary analysis of a previously published RCT [49] which was also included in this review [50].

### Participant characteristics

A total of 812 participants were included in the 23 included studies (Experimental = 404; Control = 408; Table 1), including 296 (36%) females. One study included a paediatric population only [52], while the range of the average age of the remaining 22 studies was 22 to 52 years. Only three studies included a mixed neurological population [34, 47, 52], and the TBI-specific data was acquired from one of the study’s authors and is included in this review [34]. Time post-TBI of the participants in the included studies ranged from 56 days (median) to 16.6 years (mean).

A measure of TBI severity was reported in 12 (52%) of the included studies. Injury severity was measured using the GCS [40, 53–55], length of PTA [36, 38, 40, 49, 51, 53–55], and length of loss of consciousness [36, 55]. Thirteen studies reported on the number of participants with moderate (*n* = 34) and severe (*n* = 300) TBI included in the research. The corresponding authors of the other 10 studies confirmed that all, or the majority, of participants in these studies were moderate-to-severe TBI.

### Intervention characteristics

A range of physical activity interventions were evaluated in rehabilitation (*n* = 12 studies), community (*n* = 8) and home (*n* = 3) settings (Table 2). These included structured gait/balance/functional exercise (*n* = 12 studies), structured multicomponent exercise (*n* = 5), structured aerobic training (*n* = 2), sport and physical recreation (*n* = 2) and promoting overall physical activity (*n* = 2). The length of the interventions in the included studies ranged from four to 14 weeks (mean = 8 weeks). The frequency of the interventions ranged from one to seven times per week, and the duration of the exercise sessions ranged from 15 to 90 min. The interventions in the included studies were most prescribed as individual training, with eight studies delivering the intervention as group training [37, 38, 43–45, 48, 51, 53]. All interventions included some amount of supervision, with physiotherapists most commonly providing the supervision.

**Table 2:** Intervention and outcome characteristics

Reference	PA Classification	PA Type	Supervised (by whom)	Intensity	Dose Parameters	Group Training (Size)	Location	Progression
Inpatient Rehabilitation								
Bateman et al. 2001 [34]	Structured Aerobic Exercise	Cycle Ergometer	Yes (Physiotherapist)	60–80% age predicted HR_max_	30-min, 3*/week, 12 weeks	No	Four regional neurologic inpatient rehabilitation units	Exercise time was increased as individually tolerated until the patients were able to cycle for 30-min. Work rate (watts) was then adjusted to raise each individual’s HR into a training zone: 60–80% of age-predicted HR_max_
Brown et al. 2005 [39]	Structured Gait/ Balance/ Functional Exercise	Body-Weight Supported Treadmill Training	Yes (Physiotherapist)	30% BWS 0.2–2.3 miles/hr speed	15-min, 2*/week, 14 weeks	No	NR	Reduction in BWS by 10% until 10% BWS was achieved, then reduction by 5%. All reductions were based on achieving 10 consecutive heel strikes bilaterally. Treadmill speed was increased as tolerated.
Canning et al. 2003 [40]	Structured Gait/ Balance/ Functional Exercise	Repetitive Sit-to-Stands and step-up exercises	Yes (Physiotherapist)	NR	5*/week, 4 weeks (duration NR)	No	Brain Injury Rehabilitation Unit	Increased complexity by multitasking i.e., holding cup of water. Increased speed, lowering of chair from 110–90% of lower leg length by week four. Aim was to complete 100 sit-to-stand repetitions and 60 step-ups daily, 5*days/week.
Curcio et al. 2020 [41]	Structured Gait/ Balance/ Functional Exercise	Aquatic therapy targeting enhanced postural stability and gait exercises	Yes (Physiotherapist)	NR	45-min, 3*/week, 4 weeks	NR	Hydro pool, Neurorehabilitation hospital	NR
Cuthbert et al. 2014 [42]	Structured Gait/ Balance/ Functional Exercise	VR-based balance therapy (Nintendo Wii)	Yes (Physiotherapist)	NR	15-min, 4*/week, 4 weeks	No	Hospital Gym	NR
Freivogel et al. 2009 [47]	Structured Gait/ Balance/ Functional Exercise	Robotic Body-Weight Supported Treadmill Training (LokoHelp)	Yes (Physiotherapist)	Maximal treadmill speed tolerable by participant	30-min, 3–5*/week, 6 weeks	No	Inpatient Hospital gym	Initial BWS ranged from 10-30% and was reduced as soon as possible
Wilson et al. 2006 [58]	Structured Gait/ Balance/ Functional Exercise	Partial Body-Weight Supported gait training	Yes (Physiotherapists and assistants)	NR	Max 60-min, 2*/week, 8 weeks	No	Hospital gym	% BWS reduced when participant showed no sign of discomfort with walking pace. Treadmill speed also increased when no assistance was needed, and participant showed no signs of discomfort or discoordination due to walking pace.
**Outpatient Rehabilitation**								
Esquenazi et al. 2013 [46]	Structured Gait/ Balance/ Functional Exercise	Robotic Body Weight Supported Treadmill Training	Yes (Physiotherapist)	Based on Self-Selected Velocity and/or Maximum Velocity at 10–20% BWS	60-75-min, 3*/week, 6–8 weeks	No	NR	After every 3rd training session: If either self-selected velocity or maximum velocity increased by at least 10% compared with the last assessment, the training speed increased by 10%; otherwise, training speed increased by the greater (%) of the two. If a decrease in either self-selected velocity or maximum velocity occurred, no change in training speed was implemented.
Kleffelgaard et al. 2019 [53]	Structured Gait/ Balance/ Functional Exercise	Vestibular Rehab & PA	Yes (Physiotherapist)	NR	2*/week, 8 weeks (duration NR)	Yes (2–5)	Metropolitan University	Feedback from each patient during the group sessions and their exercise diary was used to determine the parameters of the exercises throughout the intervention period. Resolution of increased symptoms within 15-30-min after the exercise session was used as a general guideline for modification and progression of the exercises. The PA was completed at home and included individually modified exercises such as walking, biking, and skiing
Särkämö et al. 2021 [55]	Structured Gait/ Balance/ Functional Exercise	Dance-Based Rehab	Yes (Dance teacher and a Physiotherapist)	NR	60-min, 2*/week, 12 weeks	No	Specialised Rehab Centre	Exercises are progressed depending on individual progress and can be done sitting or standing, alone or supported and the difficulty level and the number and type of each exercise/movement can be adjusted.
**Community-based**								
Blake et al. 2009 [37]	Sport & Physical Recreation	Tai Chi Qigong	Yes (Qigong Instructor)	NR	60-min, 1*/week, 8 weeks	Yes (unclear)	Community Day Centre	NR
Brenner et al. 2012 [38]	Promotion Overall PA	Health and Wellness Therapy Group	Yes (Allied Health Facilitators)	NA	90-min, 1*/week, 12 weeks	Yes (7–8)	Veterans Medical Centre	NA
Driver et al. 2004 [43]	Structured Multicomponent Exercise	Aquatics programme	Yes (Instructor – qualifications not specified)	50-70% HRR	60-min, 3*/week, 8 weeks	Yes (8, but 1-on-1 instruction)	Local swimming pool	Participants wore a HR monitor throughout sessions and were instructed to stay between 50-70% of HRR
Driver et al. 2006 [44]	Structured Multicomponent Exercise	Aquatics programme	Yes (Instructor – qualifications not specified)	50-70% HRR	60-min, 3*/week, 8 weeks	Yes (9, but 1-on-1 instruction)	Local swimming pool	Participants wore a HR monitor throughout sessions and were instructed to stay between 50-70% of HRR
Driver et al. 2009 [45]	Structured Multicomponent Exercise	Aquatics programme	Yes (Instructor – qualifications not specified)	50-70% HRR	60-min, 3*/week, 8 weeks	Yes (8, but 1-on-1 instruction)	Local swimming pool^a^	Participants wore a HR monitor throughout sessions and were instructed to stay between 50-70% of HRR
Gemmell et al. 2006 [48]	Sport & Physical Recreation	Tai Chi	Yes (Tai Chi Instructor)	NR	45-min, 2*/week, 6 weeks	Yes (9)	NR	The course consisted of various Tai Chi basics, including breathing and stepping techniques and five forms from the 38-step frame.
Hassett et al. 2009 [49]	Structured Multicomponent Exercise	Aerobic and muscle strength training	Yes (Personal Trainer)	Aerobic: Moderate-intensity, symptom limited such that they were breathing hard but able to talk. Strength: 6 muscle groups targeted, 3*10 or 2*15 sets/repetitions	60-min, 3*/week, 12 weeks	No	Local fitness centre	The personal trainer determined how best to complete and progress the exercises.
McMillan et al. 2002 [54]	Structured Aerobic Exercise^b^	Physical Exercise	Yes (the 5*45-min sessions were supervised by a therapist)	NR	5*45-min sessions over 4 weeks supervised & daily independent practice (duration NR)	Unclear	NR	NR
**Home-based**								
Bellon et al. 2015 [35] & Kolakowsky-Hayner et al. 2017 [36]	Promotion Overall PA	Walking	Yes (Remote supervision by a Research Assistant/Coach)	NR	7*/week, 12 weeks (no daily time limit)	No	Participant’s home	Participants were given the goal of a 5% increase in daily steps over their individual baseline for the first week. In subsequent weeks, the daily step goal was increased by the same number of steps until the participant reached a 40% increase in week eight and maintained the 40% increase over baseline for the last four weeks of the study.
Katz-Leurer et al. 2009 [52]	Structured Gait/ Balance/ Functional Exercise	Sit-to-Stands and step- ups	Yes (Parents)	Weeks 1–2: 50% max performance; Weeks 3–6: up to 75% max performance (max performance = No. sit-to-stands and No. step-ups forward and sideward in 1-min)	15-min, 5*/week, 6 weeks	No	Participant’s home	Increase repetitions
Tefertiller et al. 2019 [57]	Structured Gait/ Balance/ Functional Exercise	VR exercise targeting balance in standing	Yes (Physiotherapist)	Basic, intermediate, and advanced	30-min, 3–4*/week, 12 weeks	No	Participant’s home	Following week six testing, exercise difficulty was updated based on Community Balance and Mobility scale stratification.
**Inpatient & Outpatient Rehab**								
Hassett et al. 2012 [51]	Structured Multicomponent Exercise	Circuit class with HR feedback	Yes (Physiotherapy undergraduate students, Physiotherapy assistants, Physiotherapists)	HR training zone was calculated as ≥ 50% HRR using the Karvonen equation	60-min, 3*/week, 2 weeks	Yes (average of 8, but up to 14)	Brain Injury Unit Gym	Supervising staff used the information from the heart rate monitor to provide encouragement regarding the intensity of exercise and to progress exercises where possible (e.g., lowering the height of the chair for the sit-to-stand station).
Straudi et al. 2017 [56]	Structured Gait/ Balance/ Functional Exercise	Video game-based exercise targeting balance in standing	Yes (Physiotherapist)	NR	60-min, 3*/week, 6 weeks	No	University Hospital	Each video game had a progression over time according to patients’ abilities and successes.

*PA* Physical activity, *HR*_*max*_ Heart rate maximum, *HR* Heart rate, *BWS* Body-weight supported, *NR* Not reported, *VR* Virtual reality, *Rehab* Rehabilitation, *NA* Not applicable, *HRR* Heart rate reserve, *Max* Maximum, *No*., Number of

^a^ In Driver et al. [45], while the location of the intervention is not explicitly stated in the article, given the intervention delivered and the previous work of Driver and colleagues [43, 44], we have determined the most likely location for the intervention in this study is the local swimming pool

^b^ Classification of intervention delivered in McMillan et al. [54] was confirmed via correspondence with author and lead author of the Hassett et al. [59] Cochrane review

### Comparator characteristics

There were nine (39%) physical activity comparators, and six (26%) non-physical activity comparator interventions in the included studies (Table 1). A wait-list or no intervention was used as a comparator in five (22%) studies, while no additional intervention (i.e., only usual rehabilitation) was applied in three (13%) studies.

### Outcome measures

Across the 23 included trials, > 80 health-related outcome measures were assessed and reported on. Most reported were measures of physical function, which included measures of mobility using a composite measure (*n* = 11 studies), walking (*n* = 7), balance (*n* = 12), a global measure of function (*n* = 3), cardiorespiratory fitness (*n* = 6), muscle strength (*n* = 2), body composition (*n* = 3), and fatigue (*n* = 4). Of the other primary outcomes, three studies measured cognition and seven studies measured quality of life. Of the secondary outcomes of interest, nine studies measured mood, four studies measured participation, and two studies measured physical activity. No studies measured comorbidities and/or mortality in people with moderate-to-severe TBI.

### Adverse events

Of the 23 included studies, nine (39%) explicitly reported whether adverse events had occurred or not [34, 39, 42, 49, 52, 53, 55, 56, 58]. In total, seven adverse events were recorded, and all were from the intervention group. One study reported the occurrence of six adverse events (three participants experienced musculoskeletal pain, one experienced visual disturbance, one experienced a restriction on social outings, and one expressed feelings of depression) [49]. In one other study, a participant experienced the re-emergence of epileptic seizures [54].

### Quality appraisal

Table 1 summarizes the quality assessment of the 23 included studies. Based on the PEDro criteria, 11 of the 23 included studies were of moderate to high methodological quality (i.e., scored ≥ 7 points) [28].

### Effects of physical activity

**Fig. 2:**
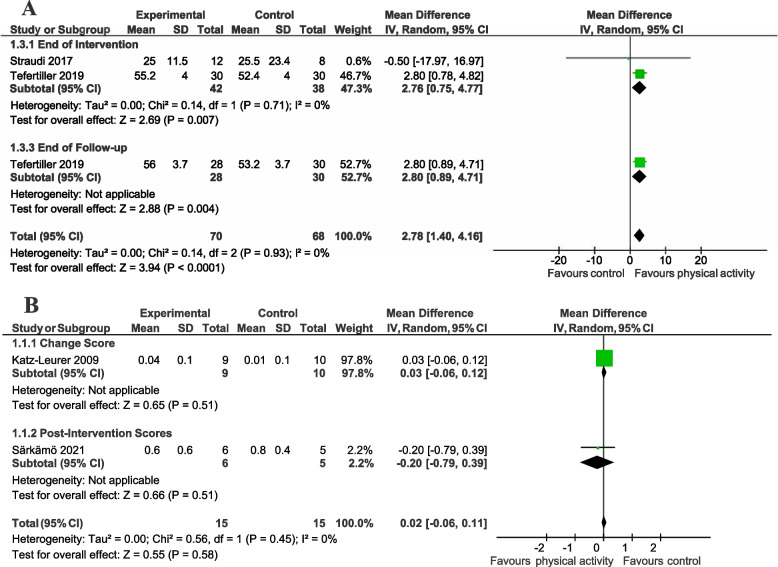
Meta-analysis of effect of a physical activity intervention on measures of composite mobility and walking. This figure presents a meta-analysis of the effect of a physical intervention vs. **A** a physical activity intervention with different parameters on a composite mobility measure; (**B**) no intervention on walking velocity

**Fig. 3:**
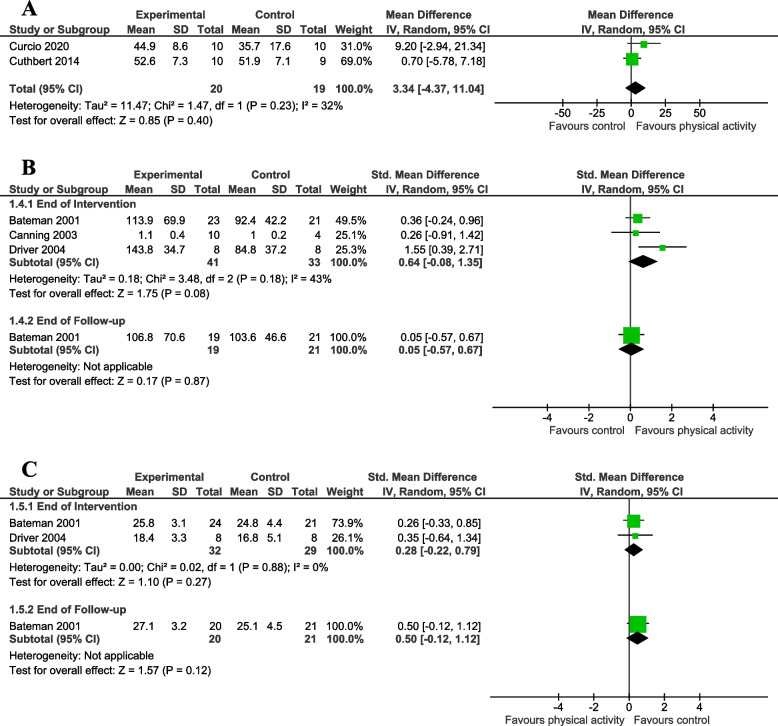
Meta-analysis of effect of physical activity intervention on balance, cardiorespiratory fitness and body composition measures. This figure presents a meta-analysis of the effect of a physical activity intervention vs. **A** a physical activity intervention with different parameters on balance; (**B**) a non-physical activity intervention or no additional intervention on cardiorespiratory fitness; (**C**) a non-physical activity intervention on body composition

**Fig. 4:**
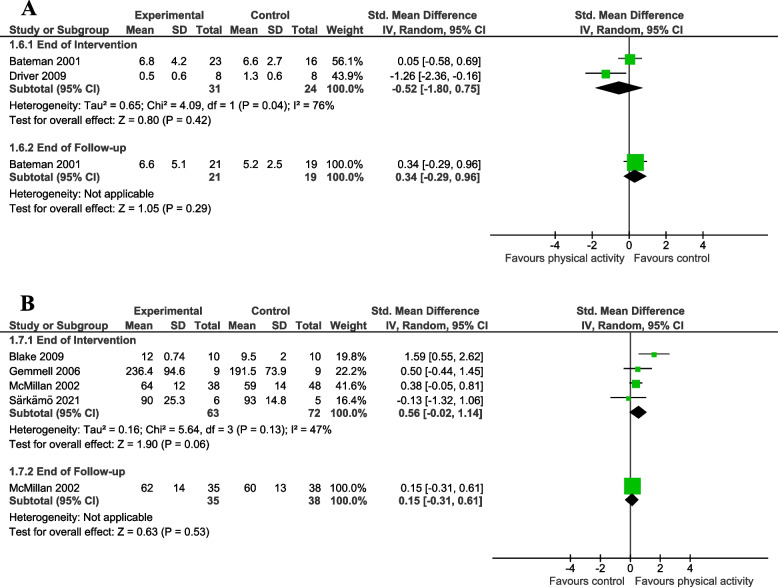
Meta-analysis of effect of physical activity intervention on measures of fatigue and quality of life. This figure presents a meta-analysis of the effect of a physical activity intervention vs. **A** a non-physical activity intervention on fatigue; (**B**) a non-physical activity intervention or no intervention on quality of life

Meta-analyses for the included outcomes are presented below and in Figs. 2, 3 and 4. We applied the GRADE criteria to rate the quality of the evidence for each of the primary outcomes (see Appendix 3 for justification for each rating). We pooled data from the end of intervention for eight outcomes (composite mobility, walking speed, balance, cardiorespiratory fitness, body composition, fatigue, quality of life, and mood). For six of the eight pooled outcomes (cardiorespiratory fitness, body composition, fatigue, quality of life, and mood), different studies used different outcome measures.

Differences between the comparison interventions in the included studies and the reporting of inconsistent data meant that for all outcome measures pooled, not all studies could be included in meta-analysis. A decision was also made to not pool primary outcomes together for meta-analysis due to the heterogeneity, including diverse interventions and comparators, and risk of bias (Appendix 3) of the included studies. Data from studies not included in meta-analysis are described in Appendix 4. There was no clear effect of physical activity on these outcomes.

The remaining outcomes (i.e., global function, other mobility, muscle strength, cognition, physical activity, and participation) were not pooled due to too many single study outcomes, the absence of data reported for the outcome, and the considerable heterogeneity among the included studies. In studies that measured global function [34, 41, 58], muscle strength, [43, 52], cognition [54–56], and physical activity [36, 37], there was no clear effect of physical activity on these outcomes (Appendix 4).

Two studies measured *mobility* by number of sit-to-stand repetitions at end of intervention [40, 52]. Significant improvements in sit-to-stand performance were found in the experimental groups. Four studies measured *participation* at end of intervention [37, 38, 49, 57], and three studies measured participation at end of follow-up [38, 49, 57]. In one study, the experimental group was significantly more successful than the control group at achieving the intervention goals (by percentage) at end of intervention [49]. The data for these outcome measures are described in Appendix 4.

## Meta-analysis

### Effect of physical activity on physical function, cognition and quality of life (primary objective)

#### Physical function

##### Composite mobility measures

We pooled the immediate effect of intervention on Community Balance and Mobility Scale (range 0 to 96; higher score indicates better mobility) data from two studies [56, 57]. The meta-analysis showed that participants randomised to virtual reality exercise improved their mobility compared to usual balance training control participants (two studies, 80 participants; MD = 2.76; 95% CI 0.75 to 4.77; low certainty evidence; Fig. 2A). One study also measured mobility at end of follow-up [57]. There appeared to be a favourable effect of the intervention on mobility maintained at end of follow-up (one study, 58 participants; MD = 2.80, 95% CI 0.89 to 4.71; Fig. 2A).

##### Walking

We pooled the immediate effect of intervention on walking speed from two studies [52, 55]. One study measured walking at end of follow-up [52]. We pooled the change scores (baseline to post-intervention) [52] and end of intervention scores [55] for analysis but present the studies as two subgroups [30]. The meta-analysis indicated there was no clear indication that participants randomised to physical activity improved their walking speed compared to control participants, with the confidence intervals indicating uncertainty about the estimate of effect (two studies, 30 participants; MD = 0.02 m/s; 95% CI -0.06 to 0.11; low certainty evidence; Fig. 2B).

##### Balance

We pooled the immediate effect of intervention Berg Balance Scale data (range 0 to 56, higher score indicates better mobility) from two studies [41, 42]. The standard error data reported in one study were converted into SD for comparison analysis [61]. The meta-analysis indicated that participants allocated to physical activity improved their balance compared to control participants, though the confidence intervals indicate uncertainty and suggest imprecision around the estimate of effect (two studies, 39 participants; MD = 3.34; 95% CI -4.37 to 11.04; I^2^ = 32%; low certainty evidence; Fig. 3A).

##### Cardiorespiratory fitness

For three studies, we pooled the immediate effect of intervention cardiorespiratory fitness data (power output at the end of a cycle ergometer test [34, 43], and peak oxygen uptake during a 3-minute maximal workload test [40]). The meta-analysis indicated participants allocated to physical activity improved cardiorespiratory fitness compared to control participants, though the confidence intervals indicate uncertainty and suggest imprecision around the estimate of effect (three studies, 74 participants; SMD = 0.64; 95% CI -0.08 to 1.35; I^2^ = 43%; low certainty evidence; Fig. 3B). One study also measured power output at end of follow-up [34]. There was no clear effect of fitness training on cardiorespiratory fitness at end of follow-up (one study, 40 participants; SMD = 0.05, 95% CI -0.57 to 0.67; Fig. 3B).

##### Body composition

We pooled the immediate effect of intervention for body mass index [34] and percentage of body fat [43] data. The meta-analysis indicated a small effect size in favour of the control intervention, though the confidence intervals indicate uncertainty and suggest imprecision around the estimate of effect (two studies, 61 participants; SMD = 0.28, 95% CI -0.22 to 0.79; low quality evidence; Fig. 3C). There was no clear effect of physical activity on body composition at end of follow-up (one study, 41 participants; SMD = 0.50, 95% CI -0.12 to 1.12; Fig. 3C).

##### Fatigue

We pooled the immediate effect of intervention for the Physical Fatigue subscale of the Chalder Fatigue Scale [34] and the fatigue subscale of the Profile of Moods State [45] data. There was an indication of a moderate reduction in self-reported fatigue with physical activity compared to a non-physical activity intervention, though the confidence intervals indicate uncertainty and suggest imprecision around the estimate of effect (two studies, 55 participants; SMD = -0.52, 95% CI -1.80 to 0.75; I^2^ = 76%; very low quality evidence; Fig. 4A). There was no clear effect of physical activity on physical fatigue at end of follow-up (one study, 40 participants; SMD = 0.34, 95% CI -0.29 to 0.96; Fig. 4A) given the confidence intervals indicate uncertainty and suggest imprecision around the estimate of effect.

### Quality of life

Two studies used the General Health Questionnaire as the outcome measure, which give a higher score for a worse outcome [37, 54]. To match the other three studies [44, 48, 55], where a higher score equals a better outcome, we subtracted the mean scores for each group from the maximum possible score for this outcome measure. For one study [48], we used only the physical summary scale of the Medical Outcomes Study Short Form-36 in the analysis.

There was substantial heterogeneity for this outcome (I^2^ = 91%; *P* < 0.01). This is likely explained by one study [44] which found quality of life was rated as significantly better in the intervention group than the control group at end of intervention (one study, 18 participants; SMD = 25.86, 95% CI 16.26 to 35.46). By excluding this study from meta-analysis, we were able to pool the remaining data (four studies, 135 participants; SMD = 0.56, 95% CI -0.02 to 1.14; I^2^ = 47%; low quality evidence; Fig. 4B). There was an indication of an improvement in quality of life for participants randomised to a physical activity intervention compared to those randomised to a control intervention at end of intervention (Fig. 4B). There is little evidence to suggest this effect was maintained at end of follow-up (one study, 73 participants; SMD = 0.15, 95% CI -0.31 to 0.61; Fig. 4B) given the confidence intervals indicate uncertainty and suggest imprecision around the estimate of effect.

### Effect of physical activity on mood (i.e., depression) (secondary objective)

#### Mood

**Fig. 5:**
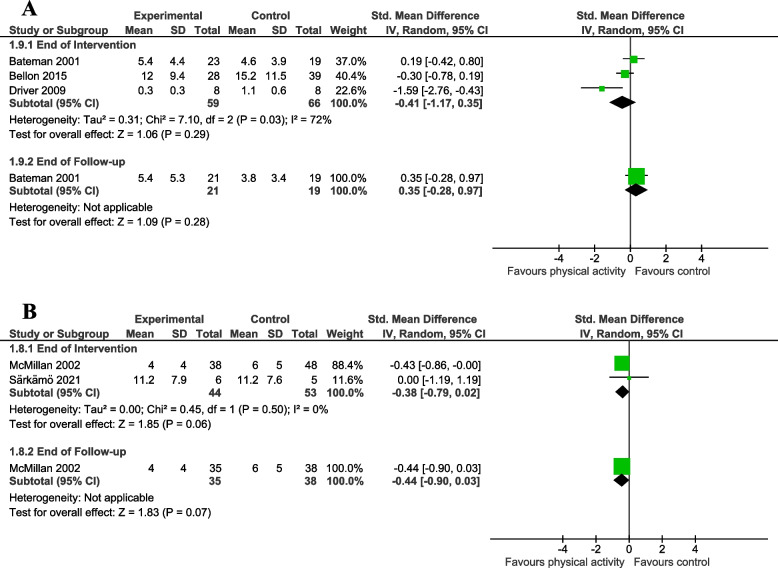
Meta-analysis of the effect of a physical activity intervention on measures of mood. This figure presents a meta-analysis of the effect of a physical activity intervention vs. **A** a non-physical activity intervention on mood; (**B**) no intervention on mood

We pooled data from three studies comparing physical activity to non-physical activity control interventions [34, 35, 45]. There was a small to moderate reduction in self-reported depression, though the confidence intervals indicate uncertainty and suggest imprecision around the estimate of effect (three studies, 125 participants; SMD = -0.41, 95% CI -1.17 to 0.35; I^2^ = 72%; Fig. 5A). There was no clear effect of physical activity at end of follow-up (one study, 40 participants; SMD = 0.35, 95% CI -0.28 to 0.97; Fig. 5A).

We also pooled data from two studies comparing physical activity to no control intervention [54, 55]. There was a small to moderate reduction in self-reported depression, though the confidence intervals indicate uncertainty and suggest imprecision around the estimate of effect (two studies, 97 participants; SMD = -0.38, 95% CI -0.79 to 0.02; I^2^ = 0%; Fig. 5B). There was no clear effect of physical activity at end of follow-up (one study, 73 participants; SMD = -0.44, 95% CI -0.90 to 0.03; Fig. 5B).

## Discussion

The primary objective of this rapid systematic review was to investigate the effect of physical activity on physical function, cognition, and quality of life across the lifespan and continuum of care for people living with moderate-to-severe TBI. We included 23 studies that covered the broad spectrum of care (i.e., inpatient, outpatient, community and home-based settings) and a wide range of physical activity interventions. For the primary outcomes of interest in this review, we were able to pool some of the available data and conduct seven meta-analyses to determine the effect of physical activity on the outcome compared to the control intervention. The results indicate an uncertainty of effect of physical activity on measures of mobility, including walking speed and balance, cardiorespiratory fitness, fatigue and quality of life in people with moderate-to-severe TBI at end of intervention. There is also little evidence of any observed improvements being maintained at follow-up. Despite more than half of the included studies being of moderate to high quality, the included studies are characterised by small sample sizes, diverse comparators and a wide range of outcome measures, including numerous single study outcomes. We are therefore unable to draw any definitive conclusions regarding the effect of physical activity on physical function, cognition and quality of life for people with moderate-to-severe TBI.

The secondary objectives of this review were to assess the effect of physical activity on mortality, comorbid conditions, mood (i.e., depression), participation and levels of physical activity. Only measures of mood data could be pooled for analysis, which showed some indication of effectiveness of physical activity. Though the confidence intervals indicate uncertainty and suggest imprecision around the estimate of effect. No studies reported on measures of comorbidity and/or mortality, while participation and physical activity was measured in only four and two studies, respectively. Again, a lack of data limits any conclusions that might be drawn from the current evidence base.

This study also aimed to evaluate the safety of physical activity interventions for people with moderate-to-severe TBI. Less than 40% of the included studies explicitly reported whether adverse events had occurred or not. The low number of reported adverse events (seven in total) and no reported serious adverse events, suggests physical activity is a safe intervention for people with moderate-to-severe TBI. Strategies to minimise the risk of harm were frequent in the included studies. All interventions in the included studies included some amount of supervision, and in six studies heart rate monitors were used to gauge effort during the training sessions and support adherence to the training protocol. In 10 studies, suitability to exercise was assessed as a part of the pre-intervention screening process, while one study required a minimum level of balance as a safety measure.

The small sample sizes and wide range of outcome measures in the included studies in this review limit our interpretation and understanding of the impact of physical activity on the health of people with moderate-to-severe TBI. It also highlights the challenges faced in research in this space and the need for a more cohesive approach moving forward. While we acknowledge the difficulties of participant recruitment in trials including moderate-to-severe TBI participants, we echo the call by Hassett et al. [59] for more adequately powered studies across the lifespan that incorporate health outcome measures framed by the International Classification of Functioning, Disability and Health (ICF) framework [62]. Identifying and using an agreed-upon core set of trial measures, with the ICF framework as a starting point for selection, would be one important step towards harmonising what is currently a disparate body of evidence. A common set of outcome measures of psychosocial function are already established in moderate-to-severe adult [63] and paediatric [64] TBI. A consensus of core physical outcome measures would further improve our ability to compare results across trials, pool data for meta-analyses or undertake individual meta-analyses, as suggested for stroke research by the Stroke Recovery and Rehabilitation Roundtable [65]. We also recommend increased collaboration between brain injury services and researchers internationally to enhance our collective capacity to recruit sufficiently powered sample sizes to answer key questions of interest. Such steps will consolidate current knowledge and facilitate optimised, evidence-based care for people with TBI in an approach aligned with AUS-TBI, an Australian-based, health informatics initiative aiming to leverage large-scale data resource to individualise care and treatment for people with TBI [66].

We acknowledge the limitations of this work, including only studies published in English. There was a limited range of participant ages included in this review – only one study included a paediatric population [52], and the average age of the remaining 22 studies was 22 to 52 years. The average sample size of all included studies was 35, ranging from 11 to 95. The small sample sizes may reduce the power of the studies included in this review; therefore, pooled meta-analyses were completed. The heterogeneity of the included studies is high. Data synthesis and reporting in this rapid review was challenging because of the variability in, and reporting of, the interventions, comparators and various outcome measures used in the included studies. For this reason, we chose not to pool primary outcomes together for meta-analysis. A standardized approach to rehabilitation trial design, delivery, and reporting, is urgently needed. We recommend future research use reporting templates, such as the CONsolidated Standards of Reporting Trials (CONSORT) statement, when reporting trials.

## Conclusion

This review was initiated in response to the WHO first global physical activity and sedentary behaviour guidelines for children and adults living with disability [8], but which did not include TBI participants or rehabilitation-based interventions. The WHO guidelines provide high-quality evidence for the beneficial effects of physical activity, and clinicians should be guided by such guidelines when prescribing physical activity. For people with TBI in rehabilitation, clinicians should be guided by evidence found here in TBI, as well as indirect evidence from other neurological populations where the evidence-base is more extensive and certain. For example, people living with moderate-to-severe TBI share similar cognitive, behavioural, and physical impairments with stroke (though people with stroke tend to be older), and cerebral palsy. This review consolidates the current evidence base for the prescription of physical activity for people with moderate-to-severe TBI. There remains a pressing need for further rigorous research to inform the development of clinical practice guidelines to support clinical decision-making when prescribing physical activity to people with TBI.

## Supplementary Information

**Additional file 1:** **Appendix 1.** PRISMA 2020 Checklist.

**Additional file 2:** **Appendix 2.** Rapid Review Search Strategies.

**Additional file 3:** **Appendix 3.** Justification of GRADE ratings.

**Additional file 4:** **Appendix 4.** Descriptive data.

## Data Availability

All extracted data used in this review has been reported in the text, figures and tables (including Appendices).

## Abbreviations

WHO: World Health Organization
TBI: Traumatic Brain Injury
PRISMA: Preferred Reporting Items for Systematic Reviews and Meta-Analyses
RCT: Randomized Controlled Trial
PTA: Post-Traumatic Amnesia
GCS: Glasgow Coma Scale
PEDRO: Physiotherapy Evidence Database
GRADE: Grading of Recommendations Assessment, Development and Evaluation
ICF: International Classification of Functioning, Disability and Health
CONSORT: Consolidated Standard of Reporting Trials
