# Correction to: the effect of physical activity on health outcomes in people with moderate-to-severe traumatic brain injury: a rapid systematic review with meta-analysis

**DOI:** 10.1186/s12889-023-15240-7

**Published:** 2023-03-06

**Authors:** Liam Johnson, Gavin Williams, Catherine Sherrington, Kavya Pilli, Sakina Chagpar, Aylish Auchett, Jack Beard, Renee Gil, Gabrielle Vassallo, Nick Rushworth, Sean Tweedy, Grahame Simpson, Adam Scheinberg, Kelly Clanchy, Anne Tiedemann, Leanne Hassett

**Affiliations:** 1 School of Health Sciences, Faculty of Medicine, Dentistry and Health Sciences, University of Melbourne, Melbourne, Australia; 2 School of Behavioural and Health Sciences, Faculty of Health Sciences, Australian Catholic University, Melbourne, Australia; 3 Physiotherapy Department, Epworth HealthCare, Melbourne, Australia; 4 Sydney School of Public Health, Faculty of Medicine and Health, The University of Sydney, Sydney, Australia; 5 Institute for Musculoskeletal Health, The University of Sydney and Sydney Local Health District, Sydney, Australia; 6 Liverpool Brain Injury Rehabilitation Unit, South Western Sydney Local Health District, Sydney, Australia; 7 Consumer representative, Sydney, Australia; 8 Brain Injury Australia, Sydney, Australia; 9 School of Human Movement and Nutrition Sciences, Faculty of Health and Behavioural Sciences, University of Queensland, Brisbane, Australia; 10 Walsh Centre for Rehabilitation Research, Kolling Institute, Northern Sydney Local Health District, The University of Sydney and Northern Sydney Local Health District, Sydney, Australia; 11 Children’s Research Institute, Melbourne, Australia; 12 School of Medicine, Faculty of Medicine, Dentistry and Health Sciences, University of Melbourne, Melbourne, Australia; 13 School of Health Sciences and Social Work, Grifth Health, Grifth University, Gold Coast, Australia; 14 Menzies Health Institute of Queensland, Grifth University, Gold Coast, Australia; 15 School of Health Sciences, Faculty of Medicine and Health, The University of Sydney, Sydney, Australia; 16 University of Sydney, Susan Wakil Health Building, D19 Western Ave, 2006 Camperdown, NSW Australia

**Correction to**: ***BMC Public Health*23, 63 (2023)**10.1186/s12889-022-14935-7

The original publication of this article contained 2 errors in Table 1 related to the PEDro scores. As a result, several other sentences of the article are affected. The correct and incorrect information is shown below. The original article has been updated. This does not affect the conclusions of the article.

**Table 1:** **Table 1 (page 5)** **Incorrect**

Reference	PEDro Quality Assessment for RCTs (total)
Straudi et al. 2017 [56]l	**7**
Tefertiller et al. 2019 [57]	**7**

**Table 2:** **Table 1 (page 5)** **correct**

Reference	PEDro Quality Assessment for RCTs (total)
Straudi et al. 2017 [56]l	**5**
Tefertiller et al. 2019 [57]	**5**

## Quality appraisal (page 7)

**Incorrect**

Based on the PEDro criteria, **11** of the 23 included studies were of moderate to high methodological quality (i.e., scored ≥ 7 points) [28].

**Correct**

Based on the PEDro criteria, **9** of the 23 included studies were of moderate to high methodological quality (i.e., scored ≥ 7 points) [28].

**Incorrect**

Despite more than half of the included studies being of moderate to high quality, the included studies are characterised by small sample sizes, diverse comparators and a wide range of outcome measures, including numerous single study outcomes.

**Correct**

Less than half of the included studies were of moderate to high quality, and the included studies are characterised by small sample sizes, diverse comparators and a wide range of outcome measures, including numerous single study outcomes.

