# Supplementary material for: The effect of physical activity on health outcomes in people with moderate-to-severe traumatic brain injury: a rapid systematic review with meta-analysis
Source: BMC Public Health. 2023 Jan 9;23:63. doi: 10.1186/s12889-022-14935-7 (PMC9830875; doi:10.1186/s12889-022-14935-7)
Supplement: Supplementary file 2 — Additional file 2: Appendix 2. Rapid Review Search Strategies. [file 12889_2022_14935_MOESM2_ESM.docx]

| **Ovid MEDLINE** | |
| --- | --- |
| **#** | **Search Statement** |
| 1 | exp Craniocerebral Trauma/ |
| 2 | Craniocerebral Trauma*.mp. |
| 3 | exp Brain Injuries, Traumatic/ |
| 4 | Diffuse Axonal Injury/ |
| 5 | diffus* axonal injur*.mp. |
| 6 | Brain Injuries, Diffuse/ |
| 7 | Diffus* brain injur*.mp. |
| 8 | exp Brain Injuries/ |
| 9 | ((head or crani* or capitis or brain* or forebrain* or skull* or hemisphere or intracran* or orbit* or cerebr*) adj1 (injur* or trauma* or lesion* or damage* or wound* or destruction* or oedema* or edema* or fracture* or contusion* or commotion* or pressur*)).tw. |
| 10 | TBI.tw. |
| 11 | 1 or 2 or 3 or 4 or 5 or 6 or 7 or 8 or 9 or 10 |
| 12 | exp Exercise/ |
| 13 | Exercis*.mp. |
| 14 | Physical activit*.mp. |
| 15 | exp Exercise Therapy/ |
| 16 | exp Physical Fitness/ |
| 17 | Physical fitness*.mp. |
| 18 | exp Sports/ |
| 19 | Sport*.mp. |
| 20 | Physical Exertion/ |
| 21 | Physical exertion.mp. |
| 22 | exp Physical Therapy Modalities/ |
| 23 | Physical therap*.mp. |
| 24 | Resistance Training/ |
| 25 | Resistance train*.mp. |
| 26 | ((exercis* or circuit or aerobic or cardio* or musc* or weight* or strength* or resistance or balance or endurance or treadmill or motor* or power* or task* or mobility or gait or fitness or physical*) adj1 (therap* or train* or retrain* or program* or intervention* or protocol* or activit* or regim* or group* or class*)).tw. |
| 27 | Physiotherap*.mp. |
| 28 | (Baseball or Basketball or Bicycl* or Boxing or Football or Golf or Gymnastics or Hockey or dance* or racquet Sport* or cricket* or team sport* or run* or skat* or snow sport* or soccer* or swim* or mountain bik* or AFL or alpine ski* or archery or athletic* or badminton or basketball or biathlon or biking or Boxing or canoe* or cricket or cross country ski* or curling or cycl* or diving or duathlon or equestrian or fencing or football or golf or gymnastics or Handball or hippotherapy or Hockey or horseback riding or horse riding or judo or kayak or kickboxing or lawn bowls or bowling or marathon or netball or badminton or snowboard or triathlon or Polo or powerlifting or rowing or sailing or shooting or skiing or snowboard or soccer or surfing or table tennis or taekwondo or Tae Kwon Do or tenpin bowling or Tennis or Trampolin* or triathlon or volleyball or volley or australian football or baseball or fencing or racing or rugby or sport* or tennis or union or league or Yoga or Tai chi or Tai ji or Chi kung or Qiqong or stretching).tw. |
| 29 | Water Sports/ |
| 30 | Racquet Sports/ |
| 31 | Snow Sports/ |
| 32 | Team sports/ |
| 33 | Return to Sport/ |
| 34 | "Return to sport".mp. |
| 35 | 12 or 13 or 14 or 15 or 16 or 17 or 18 or 19 or 20 or 21 or 22 or 23 or 24 or 25 or 26 or 27 or 28 or 29 or 30 or 31 or 32 or 33 or 34 |
| 36 | 11 and 35 |
| 37 | exp clinical trial/ |
| 38 | exp Placebos/ |
| 39 | Random Assignment*.mp. |
| 40 | control groups/ |
| 41 | cross-over studies/ |
| 42 | clinical trial.pt. |
| 43 | (crossover or cross-over or cross over).tw. |
| 44 | (random* or placebo* or "clinical trial*").mp. |
| 45 | ((singl* or doubl* or trebl* or tripl*) adj1 (blind* or mask*)).ab,ti. |
| 46 | Systematic review.tw,pt. |
| 47 | exp Systematic review/ |
| 48 | Systematic review*.tw,pt. |
| 49 | exp Randomized Controlled Trials as Topic/ |
| 50 | (randomized controlled trial or controlled clinical trial or placebo*).tw,pt. |
| 51 | Meta-Analysis/ |
| 52 | (meta-analys* or metaanalys* or meta analys*).ab,ti |
| 53 | 37 or 38 or 39 or 40 or 41 or 42 or 43 or 44 or 45 or 46 or 47 or 48 or 49 or 50 or 51 or 52 |
| 54 | 36 and 53 |
| 55 | limit 54 to humans |

| **CENTRAL via Ovid** | |
| --- | --- |
| **#** | **Search Statement** |
| 1 | MeSH descriptor: [Craniocerebral Trauma] explode all trees |
| 2 | (Craniocerebral Trauma*) |
| 3 | MeSH descriptor: [Brain Injuries, Traumatic] explode all trees |
| 4 | MeSH descriptor: [Diffuse Axonal Injury] explode all trees |
| 5 | (diffus* axonal injur*) |
| 6 | MeSH descriptor: [Brain Injuries, Diffuse] explode all trees |
| 7 | ((head or crani* or capitis or brain* or forebrain* or skull* or hemisphere or intracran* or orbit* or cerebr*) NEAR/1 (injur* or trauma* or lesion* or damage* or wound* or destruction* or oedema* or edema* or fracture* or contusion* or commotion* or pressur*)):ti,ab,kw |
| 8 | (Diffus* brain injur*) |
| 9 | (TBI):ti,ab,kw |
| 10 | MeSH descriptor: [Brain Injuries] explode all trees |
| 11 | #1 OR #2 OR #3 OR #4 OR #5 OR #6 OR #7 OR #8 OR #9 OR #10 |
| 12 | MeSH descriptor: [Exercise] explode all trees |
| 13 | (Exercis*) |
| 14 | (Physical activit*) |
| 15 | MeSH descriptor: [Exercise Therapy] explode all trees |
| 16 | MeSH descriptor: [Physical Fitness] explode all trees |
| 17 | (Physical fitness*) |
| 18 | MeSH descriptor: [Sports] explode all trees |
| 19 | (Sport*) |
| 20 | MeSH descriptor: [Physical Exertion] explode all trees |
| 21 | (physical exertion) |
| 22 | MeSH descriptor: [Physical Therapy Modalities] explode all trees |
| 23 | (Physical therap*) |
| 24 | MeSH descriptor: [Resistance Training] explode all trees |
| 25 | (Resistance train*) |
| 26 | (Physiotherap*) |
| 27 | (((exercis* or circuit or aerobic or cardio* or musc* or weight* or strength* or resistance or balance or endurance or treadmill or motor* or power* or task* or mobility or gait or fitness or physical*) NEAR/1 (therap* or train* or retrain* or program* or intervention* or protocol* or activit* or regim* or group* or class*))):ti,ab,kw |
| 28 | ((Baseball or Basketball or Bicycl* or Boxing or Football or Golf or Gymnastics or Hockey or dance* or racquet Sport* or cricket* or team sport* or run* or skat* or snow sport* or soccer* or swim* or mountain bik* or AFL or alpine ski* or archery or athletic* or badminton or basketball or biathlon or biking or Boxing or canoe* or cricket or cross country ski* or curling or cycl* or diving or duathlon or equestrian or fencing or football or golf or gymnastics or Handball or hippotherapy or Hockey or horseback riding or horse riding or judo or kayak or kickboxing or lawn bowls or bowling or marathon or netball or badminton or snowboard or triathlon or Polo or powerlifting or rowing or sailing or shooting or skiing or snowboard or soccer or surfing or table tennis or taekwondo or Tae Kwon Do or tenpin bowling or Tennis or Trampolin* or triathlon or volleyball or volley or australian football or baseball or fencing or racing or rugby or sport* or tennis or union or league or Yoga or Tai chi or Tai ji or Chi kung or Qiqong or stretching)):ti,ab,kw |
| 29 | MeSH descriptor: [Water Sports] explode all trees |
| 30 | MeSH descriptor: [Racquet Sports] explode all trees |
| 31 | MeSH descriptor: [Snow Sports] explode all trees |
| 32 | MeSH descriptor: [Team Sports] explode all trees |
| 33 | MeSH descriptor: [Return to Sport] explode all trees |
| 34 | (return to sport) |
| 35 | #12 OR #13 OR #14 OR #15 OR #16 OR #17 OR #18 OR #19 OR #20 OR #21 OR #22 OR #23 OR #24 OR #25 OR #26 OR #27 OR #28 OR #29 OR #30 OR #31 OR #32 OR #33 OR #34 |
| 36 | #11 AND #35 |

| **SportDiscus via EBSCO** | |
| --- | --- |
| **#** | **Search Statement** |
| S1 | "BRAIN damage" OR "BRAIN injuries" OR "BRAIN damage" OR "CHRONIC traumatic encephalopathy" |
| S2 | "diffus* axonal injur*" |
| S3 | "diffus* brain injur*" |
| S4 | ((“head” OR “crani*” OR “capitis” or “brain*” OR “forebrain*” OR “skull*” OR “hemisphere” OR “intracran*” OR “orbit*” OR “cerebr*”) N1 (“injur*” OR “trauma*” OR “lesion*” or “damage*” OR “wound*” OR “destruction*” OR “oedema*” OR “edema*” OR “fracture*” OR “contusion*” OR “commotion*” OR “pressur*”) ) |
| S5 | TI "TBI" OR AB "TBI" |
| S6 | S1 OR S2 OR S3 OR S4 OR S5 |
| S7 | exercis* |
| S8 | "Physical activit*" |
| S9 | "exercise therap*" |
| S10 | "physical fitness" |
| S11 | Sport* |
| S12 | “recreational therap*” |
| S13 | "Resistance train*" |
| S14 | ( (“exercis*” OR “circuit” OR “aerobic” OR “cardio*” OR “musc*” OR “weight*” OR “strength*” OR “resistance” OR “balance” OR “endurance” OR “treadmill” OR “motor*” OR “power*” OR “task*” OR “mobility” OR “gait” OR “fitness” OR “physical*”) N1 (“therap*” OR “train*” OR “retrain*” OR “program*” OR “intervention*” OR “protocol*” OR “activit*” OR “regim*” OR “group*” OR “class*”) ) |
| S15 | "physiotherap*" |
| S16 | ( “Baseball” OR “Basketball” OR “Bicycl*” OR “Boxing” OR “Football” OR “Golf” OR “Gymnastics” OR “Hockey” OR “dance*” OR “racquet Sport*” OR “cricket*” OR “team sport*” OR “run*” OR “skat*” OR “snow sport*” OR “soccer*” OR “swim*” OR “mountain bik*” OR “AFL” OR “alpine ski*” OR “archery” OR “athletic*” OR “badminton” OR “basketball” OR “biathlon” OR “biking” OR “Boxing” OR “canoe*” OR “cricket” OR “cross country ski*” OR “curling” OR “cycl*” OR “diving” OR “duathlon” OR “equestrian” OR “fencing” OR “football” OR “golf” OR “gymnastics” OR “Handball” OR “hippotherapy” OR “Hockey” OR “horseback riding” OR “horse riding” OR “judo” OR “kayak” OR “kickboxing” OR “lawn bowls” OR “bowling” OR “marathon” OR “netball” OR “badminton” OR “snowboard” OR “triathlon” OR “Polo” OR “powerlifting” OR “rowing” OR “sailing” OR “shooting” OR “skiing” OR “snowboard*” OR “soccer” OR “surfing” OR “table tennis” OR “taekwondo” OR “Tae Kwon Do” OR “tenpin bowling” OR “Tennis” OR “Trampolin*” OR “triathlon” OR “volleyball” OR “volley” OR “australian football” OR “baseball” OR “fencing” OR “racing” OR “rugby” OR “sport*” OR “tennis” OR “union “OR “league” OR “Yoga” OR “Tai chi” OR “Tai ji” OR “Chi kung” OR “Qiqong” OR “stretching” or "team sport* or "ball game*" or "Aquatic sport*" or "water sport*" or "racquet sport*" or "snow sport*" or "team sport*" or "return to sport*" ) |
| S17 | hydrotherap* |
| S18 | S8 OR S9 OR S10 OR S11 OR S12 OR S13 OR S14 OR S15 OR S16 OR S17 |
| S19 | TI ( ("singl*" OR "doubl*" OR "trebl*" OR "tripl*") N1 ("blind*" OR "mask*") ) OR AB ( ("singl*" OR "doubl*" OR "trebl*" OR "tripl*") N1 ("blind*" OR "mask*") ) |
| S20 | TI ( "Systematic review*" OR AB "Systematic review*" ) OR AB ( TI "Systematic review*" OR AB "Systematic review*" ) |
| S21 | TI ( "randomized controlled trial" OR "controlled clinical trial" OR "placebo*" ) OR AB ( "randomized controlled trial" OR "controlled clinical trial" OR "placebo*" ) |
| S22 | TI ( meta-analys* OR metaanalys* OR meta analys* ) OR AB ( meta-analys* OR metaanalys* OR meta analys* ) |
| S23 | "controlled trial" OR "clinical trial" or random* or "random sampling" or "clinical trial* or "crossover" OR "crossover" OR "cross over" |
| S24 | S19 OR S20 OR S21 OR S22 OR S23 |
| S25 | S6 AND S18 AND S24 |

| **Physiotherapy Evidence Database (PEDro)- run offline** | |
| --- | --- |
| # | Search Statement |
| 1 | (brain or head) [Abstract & Title] AND neurotrauma [Topic] |
| 2 | (exercise* OR physical activit* OR fitness OR sport*) [Abstract & Title] OR (fitness training OR skill training OR strength training) [Therapy] |
| 3 | 1 AND 2 |
| 4 | 3 (exclude guidelines) |
