# Supplementary material for: The effect of physical activity on health outcomes in people with moderate-to-severe traumatic brain injury: a rapid systematic review with meta-analysis
Source: BMC Public Health. 2023 Jan 9;23:63. doi: 10.1186/s12889-022-14935-7 (PMC9830875; doi:10.1186/s12889-022-14935-7)
Supplement: Supplementary file 3 — Additional file 3: Appendix 3. Justification of GRADE ratings. [file 12889_2022_14935_MOESM3_ESM.docx]

Table 3. Justification of GRADE rating for pooled primary outcomes.

| **Outcome** | **Reason to downgrade the evidence** | | | | | **Reasons to upgrade the evidence** | **GRADE rating** |
| --- | --- | --- | --- | --- | --- | --- | --- |
|  | **Risk of bias of study design** | **Indirectness of evidence** | **Heterogeneity** | **Imprecision** | **Publication bias** | **Large magnitude of effect, dose response, effect of all plausible confounding factors** |  |
| Composite Mobility  (2 studies, 80 participants) | Downgraded x 1 level: unblinded assessor for 2 studies | Nil | Nil: I^2^ = 0%  Chi^2^ P = 0.71 | Downgraded x 1 level: wide CI and 1 study with positive and 1 study with negative mean differences | Nil: both studies registered as clinical trials. No bias serious enough to downgrade | Nil | Downgraded  2 levels to LOW |
| Walking  (2 studies, 30 participants) | Downgraded x 1 level: allocation concealment at high risk of bias for 1 study; unblinded participants and therapists for 1 study; unblinded therapists and assessors for 1 study | Nil | Nil: I^2^ = 0%  Chi^2^ P = 0.45 | Downgraded x 1 level: wide CI and 1 study with positive and 1 study with negative mean difference | Nil: no published  protocol, but no bias serious enough to downgrade | Nil | Downgraded  2 levels to LOW |
| Balance  (2 studies, 39 participants) | Downgraded x 1 level: unblinded participants, therapists and assessors for 1 study; unblinded therapists and assessors for 1 study | Nil | Nil: I^2^ = 32%  Chi^2^ P = 0.23 | Downgraded x 1 level: wide CI | Nil: 1 study registered as a clinical trial. No bias serious enough to downgrade | Nil | Downgraded  2 levels to LOW |
| Cardiorespira-tory fitness  (3 studies, 74 participants) | Downgraded x 1 level: allocation concealment at high risk of bias for 1 study; unblinded participants, therapists and assessors for 1 study; unblinded participants and therapists for 2 studies | Nil: not gold  standard  measure, but  still common  measure of  fitness | Nil: I^2^ = 43%  Chi^2^ P = 0.18 | Downgraded x 1 level: wide CI | Nil: no published  protocol, but no bias serious enough to downgrade | Nil | Downgraded  2 levels to LOW |
| Body composition  (2 studies, 61 participants) | Downgraded x 1 level: allocation concealment at high risk of bias for 1 study | Nil | Nil: I^2^ = 0%  Chi^2^ P = 0.88 | Downgraded x 1 level: wide CI | Nil: no published  protocol, but no bias serious enough to downgrade | Nil | Downgraded  2 levels to LOW |
| Fatigue  (2 studies, 55 participants) | Downgraded x 1 level: allocation concealment unclear for 1 study and low for 1 study; high risk (1 study) or unclear risk (1 study) of detection bias: participants not blinded for self-reported measure | Nil | Downgraded x 1 level:  Nil: I^2^ = 76%  Chi^2^ P = 0.04 | Downgraded x 1: wide CI and 1 study with negative and 1 study with positive mean difference | Nil: no published  protocol, but no bias serious enough to downgrade | Nil | Downgraded  3 levels to  VERY LOW |
| Quality of life  (4 studies, 135 participants) | Downgraded x 1 level: allocation concealment at high risk of bias for 4 studies; unblinded participants, therapists and assessors for 1 study; unblinded participants and therapists for 2 studies; unblinded assessors for 1 study | Nil | Nil: I^2^ = 47%  Chi^2^ P = 0.13 | Downgraded x 1: 1 study with negative and 3 studies with positive mean difference | Nil: no published  protocol, but no bias serious enough to downgrade | Nil | Downgraded  2 levels to LOW |

GRADE, Grading of Recommendations, Assessment, Development and Evaluations; CI, Confidence intervals.
