# Supplementary material for: The effect of physical activity on health outcomes in people with moderate-to-severe traumatic brain injury: a rapid systematic review with meta-analysis
Source: BMC Public Health. 2023 Jan 9;23:63. doi: 10.1186/s12889-022-14935-7 (PMC9830875; doi:10.1186/s12889-022-14935-7)
Supplement: Supplementary file 4 — Additional file 4: Appendix 4. Descriptive data. [file 12889_2022_14935_MOESM4_ESM.docx]

| **Outcome** | **Study** | **Outcome Measure** | **Timing** | **Intervention Group**  **Mean (SD); N** | **Control Group Mean (SD); N** | **Mean between group difference**  **(MD), 95% CI** |
| --- | --- | --- | --- | --- | --- | --- |
| Composite Mobility | Bateman et al.[34] | Rivermead Mobility Index (/15 ↑) | End of intervention | 11.2 (3.7); 24 | 12.1 (3.7); 21 | MD -0.9, 95% CI -3.1 to 1.3 |
|  |  |  | End of follow-up | 11.2 (3.6); 22 | 12.8 (3.4); 21 | MD -1.6, 95% CI -3.7 to 0.5 |
|  | Brown et al.[39] | Time-up-and-go (s) | End of intervention | 52 (41.0); 10 | 63 (0.57)*; 9 | MD -11, 95% CI -36.4 to 14.4 |
|  | Curcio et al.[41] | Tinetti Gait Balance Scale (/28 ↑) | End of intervention | 23.5 (3.7); 10 | 19.1 (8.3); 10 | MD 4.4, 95% CI -1.2 to 10.0 |
|  | Cuthbert et al.[42] | Functional Gait Assessment (/30 ↑) | End of intervention | 22.8 (6.5); 10 | 23.3 (6.3); 9 | MD -0.5, 95% CI -6.3 to 5.3 |
|  | Esquenazi et al.[46] | Stroke Impact Scale (mobility domain, 9-45 ↑)^ | End of intervention | 80.9 (NR); 8 | 78.6 (NR); 7 | Not estimable |
|  | Freivogel et al.[47] | Rivermead Mobility Index (/15 ↑) | End of intervention | NR (NR); 8 | NR (NR); 8 | Not estimable |
|  | Katz-Leurer et al.[52]⁺ | Timed-up-and-go (m/s) | End of intervention | 1.6 (2.1); 9 | 0.0 (0.7); 10 | MD 1.6, 95% CI 0.2 to 3.0 |
|  |  |  | End of follow-up | 0.0 (0.9); 8 | NR (NR); NR | Not estimable |
|  | Kleffelgaard et al.[53] | High-Level Mobility Assessment Tool (/54 ↑) | End of intervention | 47.6 (7.0); 29 | 41.2 (12.3); 23 | MD 6.4, 95% CI 0.8 to 12.0 |
|  |  |  | End of follow-up | 47.3 (8.2); 25 | 44.3 (9.6); 26 | MD 3.0, 95% CI -1.9 to 7.9 |
|  | Straudi et al.[56] | Timed-up-and-go (s) | End of intervention | 16.4 (7.0); 12 | 15.4 (12.0); 8 | MD 1.0, 95% CI -8.2 to 0.2 |
|  |  | Unified Balance Scale | End of intervention | 49.5 (15.2); 12 | 51.0 (15.2); 8 | MD -1.5, 95% CI -18.1 to 15.1 |
|  | Wilson et al.[58] | Rivermead Mobility Index (/15 ↑) | End of intervention | 7.8 (5.2); 19 | 6.9 (4.6); 19 | MD 0.9, 95% CI -2.2 to 4.0 |
| Walking | Bateman et al.[34] | Walking Velocity (cm/s) | End of intervention | 120 (85); 24 | 144 (86); 21 | MD -24, 95% CI -74.1 to 26.1 |
|  | Brown et al.[39] | Walking Velocity (cm/s) | End of intervention | 32.8 (24.1); 10 | 42 (32.5); 9 | MD -9.2, 95% CI -35.2 to 16.8 |
|  |  | Functional Ambulation Category (0-5 ↑) | End of intervention | 2.2 (1.5); 10 | 2.7 (1.6); 9 | MD -0.5, 95% CI -1.9 to 0.9 |
|  |  | Step length differential (cm) | End of intervention | 25 (25.6); 10 | 12.4 (13.2); 9 | MD 12.6, 95% CI -5.5 to 30.7 |
|  | Esquenazi et al.[46] | Walking velocity (cm/s) | End of intervention | 78 (NR); 8 | 80 (NR); 7 | Not estimable |
|  |  | Six-Minute Walk Test (m) | End of intervention | 188.3 (NR); 8 | 171.5 (NR); 7 | Not estimable |
|  |  | Step-length asymmetry ratio (cm) | End of intervention | 44 (NR); 8 | 29 (NR); 7 | Not estimable |
|  | Freivogel et al.[47] | Functional Ambulation Category (0-5 ↑) | End of intervention | NR (NR); 8 | NR (NR); 8 | Not estimable |
|  | Katz-Leurer et al.[52]⁺ | Two-Minute Walk Test (m) | End of intervention | 8 (13.5); 9 | 0.2 (22.4); 10 | MD 7.8, 95% CI -8.6 to 24.2 |
|  |  |  | End of follow-up | 1.8 (11.1); 8 | NR (NR); NR | Not estimable |
|  | Wilson et al.[58] | Functional Ambulation Category (0-5 ↑) | End of intervention | 2.8 (1.8); 19 | 2.7 (1.8); 19 | MD 0.1, 95% CI -1.0 to 1.2 |
| Balance | Brown et al.[39] | Function Reach (inches) | End of intervention | 6.2 (4.5); 10 | 5 (5.3); 9 | MD 1.2, 95% CI -3.2 to 5.6 |
|  | Driver et al.[43] | Sit-and-reach (inches) | End of intervention | 14.8 (3.8); 8 | 14.5 (7.7); 8 | MD 0.3, 95% CI -5.6 to 6.2 |
|  | Freivogel et al.[47] | Berg Balance Scale (/56 ↑) | End of intervention | NR (NR); 8 | NR (NR); 8 | Not estimable |
|  | Katz-Leurer et al.[52]⁺ | Functional Reach (forward, cm) | End of intervention | 3.0 (1.6); 9 | 1.9 (4); 10 | MD 1.1, 95% CI -1.6 to 3.8 |
|  |  |  | End of follow-up | 0.1 (2.1); 8 | NR (NR); NR | Not estimable |
|  | Kleffelgaard et al.[53] | Balance Error Scoring System (/60 ↓) | End of intervention | 19.1 (10.6); 31 | 23 (9.1); 26 | MD -3.9, 95% CI -9.0 to 1.2 |
|  |  |  | End of follow-up | 17.5 (10.4); 26 | 20.8 (9.0); 28 | MD -3.3, 95% CI -8.5 to 1.9 |
|  | Särkämö et al.[55] | Berg Balance Scale (/56 ↑) | End of intervention | 27.2 (18.5); 6 | 44 (10.8); 5 | MD -16.8, 95% CI -34.4 to 0.8 |
|  | Straudi et al.[56] | Static Balance, total path length, eyes open (mm) | End of intervention | 266.3 (129.8); 12 | 427.3 (141.3); 8 | MD -161.0, 95% CI -283.4 to -38.6 |
|  | Tefertiller et al.[57] | Balance Evaluation Systems Test (/108 ↑) | End of intervention | 84.8 (3.2); 30 | 84.2 (3.1); 30 | MD 0.6 95% CI -1.0 to 2.2 |
|  |  |  | End of follow-up | 86.3 (3.1); 28 | 84.7 (3.1); 30 | MD 1.6 95% CI 0.0 to 3.2 |
|  |  | Activities-specific Balance Confidence Scale (% ↑) | End of intervention | 83.6 (2.6); 30 | 79.5 (2.5); 30 | MD 4.1, 95% CI 2.8 to 5.4 |
|  |  |  | End of follow-up | 85.8 (2.7); 28 | 79.4 (2.7); 30 | MD 6.4, 95% CI 5.0 to 7.8 |
|  | Wilson et al.[58] | Standing Balance Score (0-4 ↑) | End of intervention | 2.6 (1.8); 19 | 2.7 (1.8); 19 | MD -0.1, 95% CI -1.2 to 1.0 |
| Other Mobility Measures | Canning et al.[40] | Sit-to-Stand Repetitions (maximum in 3-min) | End of intervention | 79.3 (33.7); 12 | 87.7 (33.9); 9 | MD -8.4, 95% CI -37.6 to 20.8 |
|  | Katz-Leurer et al.[52]⁺ | Sit-to-Stand Repetitions (maximum in 1-min) | End of intervention | 3.7 (3.5); 9 | NR (NR); 10 | Not estimable |
|  |  | Step-up Repetitions (maximum in 1-min) | End of intervention | 3.0 (2.7); 9 | NR (NR); 10 | Not estimable |
| Global Function | Bateman et al.[34] | Barthel Index (/20 ↑) | End of intervention | 17 (4); 24 | 17.9 (3.2); 22 | MD -0.9, 95% CI -3.0 to 1.2 |
|  |  |  | End of follow-up | 17.5 (4.4); 22 | 18.6 (3.1); 21 | MD -1.1, 95% CI -3.4 to 1.2 |
|  | Curcio et al.[51] | Barthel Index (modified) (/100 ↑) | End of intervention | 91.2 (6.7); 10 | 77.0 (19.0); 10 | MD 14.2, 95% CI 1.7 to 26.7 |
|  | Wilson et al.[58] | Gross Motor Subscale (/12 ↓) | End of intervention | 5.6 (4.2); 19 | 5.5 (4.4); 19 | MD 0.1, 95% CI -2.6 to 2.8 |
|  |  | Functional Independent Measure + Functional Assessment Measure (1-7 ↑) | End of intervention | 4.8 (2.2); 19 | 4.4 (2.0); 19 | MD 0.4, 95% CI -0.94 to 1.7 |
| Cardiorespiratory Fitness | Bateman et al.[34] | Peak Heart rate during submaximal graded exercise test (beats per min) | End of intervention | 158.2 (21.1); 23 | 153.7 (25.4); 21 | MD 4.5, 95% CI -9.4 to 18.4 |
|  |  |  | End of follow-up | 152.8 (28.9); 19 | 159.7 (25); 21 | MD -6.9 95% CI -23.7 to 9.9 |
|  | Canning et al.[40] | Exercise efficiency (VO_2_ equivalent) during a 3-min equivalent workload test (L.min^-1^) | End of intervention | 0.5 (0.2); 9 | 0.5 (0.1); 8 | MD 0.0, 95% CI -0.2 to 0.2 |
|  | Driver et al.[43] | Submaximal cycle ergometry time (min) | End of intervention | 9.6 (2.1); 8 | 5.4 (1.6); 8 | MD 4.2, 95% CI 2.4 to 6.0 |
|  | Hassett et al.[49] | Distance completed during 20-m shuttle test (m) | End of intervention | 695.0 (358.0); 32 | 807.0 (365.0); 30 | MD -112.0, 95% CI -292.1 to 68.1 |
|  |  |  | End of follow-up | 713.0 (375.0); 32 | 854.0 (398.0); 30 | MD -141.0, 95% CI -333.8 to 51.8 |
|  | Hassett et al.[51] | Time spent in HR training zone (i.e. ≥ 50% heart rate reserve) (min) | End of intervention | 8.3 (8.9); 20 | 7.1 (9.4); 20 | MD 1.2, 95% CI -4.5 to 6.9 |
| Muscle strength | Driver et al.[43] | Grip Strength (right, Kg) | End of intervention | 21.7 (13.4); 8 | 21.6 (13.2); 8 | MD 0.1, 95% CI -12.9 to 13.1 |
|  |  | Grip Strength (left, Kg) | End of intervention | 29.8 (15.4); 8 | 32.8 (18.1); 8 | MD -3.0, 95% CI -19.5 to 13.5 |
|  |  | Modified curl-up (repetitions) | End of intervention | 59.3 (21.7); 8 | 62.3 (26.1); 8 | MD -3.0, 95% CI -26.5 to 20.5 |
|  | Katz-Leurer et al.[52]⁺ | Isometric muscle strength – hip abduction (Kg) | End of intervention | 0.7 (1.9); 9 | 0.8 (1.1); 10 | MD -0.1, 95% CI -1.5 to 1.3 |
|  |  |  | End of follow-up | 0.3 (1.4); 8 | NR (NR); NR | Not estimable |
|  |  | Isometric muscle strength – hip extension (Kg) | End of intervention | 1.6 (2.2); 9 | 0.7 (1.3); 10 | MD 0.9, 95% CI -0.8 to 2.6 |
|  |  |  | End of follow-up | 0.6 (1.2); 8 | NR (NR); NR | Not estimable |
|  |  | Isometric muscle strength – knee extension (Kg) | End of intervention | 0.2 (4.4); 9 | 0.0 (0.7); 10 | MD O.2, 95% CI -2.7 to 3.1 |
|  |  |  | End of follow-up | 0.0 (0.8); 8 | NR (NR); NR | Not estimable |
|  |  | Isometric muscle strength – knee flexion (Kg) | End of intervention | 0.0 (3.0); 9 | 0.1 (1.0); 10 | MD -0.1, 95% CI -2.2 to 2.0 |
|  |  |  | End of follow-up | 0.3 (1.4); 8 | NR (NR); NR | Not estimable |
| Body Composition | Hassett et al.[49] | Body Mass Index (Kg/m^2^) | End of intervention | 23.9 (3.5); 15 | 23.4 (3.6); 13 | MD 0.5, 95% CI -2.1 to 3.1 |
|  |  |  | End of follow-up | 24.7 (3.8); 18 | 23.2 (3.6); 16 | MD 1.5, 95% CI -1.0 to 4.0 |
|  |  | Waist Circumference (cm) | End of intervention | 83.6 (8.7); 15 | 82.4 (10.2); 12 | MD 1.2, 95% CI -6.1 to 8.5 |
|  |  |  | End of follow-up | 84.3 (9.2); 18 | 81.1 (9.4); 17 | MD 3.2, 95% CI -3.0 to 9.4 |
|  |  | Waist-to-hip ratio | End of intervention | 0.9 (0.1); 15 | 0.9 (0.1); 17 | MD 0.0, 95% CI -0.1 to 0.1 |
|  |  |  | End of follow-up | 0.9 (0.1); 18 | 0.9 (0.1); 12 | MD 1.2, 95% CI -6.1 to 8.5 |
| Fatigue | Bateman et al.[34] | Chalder Modified Fatigue Scale (mental fatigue subscale, /10 ↓) | End of intervention | 5.3 (3.1); 23 | 4.4 (2.1); 16 | MD 0.9, 95% CI -0.7 to 2.5 |
|  |  |  | End of follow-up | 5.9 (3.8); 21 | 4.0 (2.4); 19 | MD 1.9, 95% CI -0.1 to 3.8 |
|  | Hassett et al.[49] | Profile of Moods State (fatigue subscale, *t*-score converted from raw score ↓) | End of intervention | 47.0 (7.0); 32 | 43.0 (6.0); 30 | MD 4.0, 95% CI 0.8 to 7.2 |
|  |  |  | End of follow-up | 46.0 (8.0); 32 | 43.0 (5.0); 30 | MD 3.0, 95% CI -0.3 to 6.3 |
|  | Kolakowsky-Hayner et al.[36] | Global Fatigue Index (1-50 ↓) | End of intervention | 20.5 (NR); NR | 24.6 (NR); NR | Not estimable |
|  |  | Barrow Neurological Institute Fatigue Scale (/70 ↓) | End of intervention | 19.7 (NR); NR | 24.4 (NR); NR | Not estimable |
|  |  | Multidimensional Fatigue Inventory (General Fatigue subscale 4-20 ↓) | End of intervention | 9.9 (NR); NR | 11.1 (NR); NR | Not estimable |
| Cognition | McMillan et al.[54] | Self-Reported Cognitive Failures (/100 ↓) | End of intervention | 41 (17); 38 | 38 (22); 48 | MD 3.0, 95% CI -5.2 to 11.2 |
|  |  |  | End of follow-up | 38 (17); 35 | 41 (19); 38 | MD -3.0, 95% CI -11.3 to 5.3 |
| Quality of Life | Curcio et al.[41] | Quality of Life after Brain Injury scale (unclear scoring ↑) | End of intervention | 59.4 (16.5); 10 | 62.3 (17.7); 10 | MD -2.9, 95% CI -17.9 to 12.1 |
|  | Gemmell et al.[48] | The Medical Outcomes Study Short Form-36 (mental health summary scale) (unclear scoring) | End of intervention | 206.8 (61.8); 9 | 205.2 (63.7); 9 | MD 1.6, 95% CI -56.4 to 59.6 |
| Mood | Bellon et al.[35] | Perceived Stress Scale (/56 ↓) | End of intervention | 20.8 (8.1); 29 | 24.3 (10.9); 40 | MD -3.5, 95% CI -8.0 to 1.0 |
|  | Gemmell et al.[48] | Rosenberg Self-Esteem Scale (/30 ↑) | End of intervention | 56.7 (22.2); 9 | 58.2 (18.9); 9 | MD -1.5, 95% CI -20.6 to 17.6 |
|  | Hassett et al.[49] | Depression Anxiety and Stress Scale (depression subscore) (/42 ↓) | End of intervention | 5.3 (8.2); 32 | 2.4 (3.6); 30 | MD 2.9, 95% CI -5.2 to 5.2 |
|  |  |  | End of follow-up | 5.7 (8.9); 32 | 5.7 (11.5); 30 | MD 0.0, 95% CI -0.2 to 6.0 |
|  |  | Profile of Moods State (depression/dejection subscale) (*t*-score converted from raw score ↓) | End of intervention | 41.0 (7.0); 32 | 39.0 (7.0); 30 | MD 2.0, 95% CI -1.5 to 5.5 |
|  |  |  | End of follow-up | 41.0 (6.0); 32 | 40.0 (6.0); 30 | MD 1.0, 95% CI -2.0 to 4.0 |
|  | Kleffelgaard et al.[53] | Hospital Anxiety and Depression Scale (depression subscale) (/21 ↓) | End of intervention | 6.1 (4.3); 32 | 6.2 (5.4); 31 | MD -0.1, 95% CI -2.5 to 2.3 |
|  |  |  | End of follow-up | 6.7 (5.2); 27 | 5.1 (4.8); 28 | MD 1.6, 95% CI -1.0 to 4.2 |
| Participation | Blake et al.[37] | Social Support for Exercise Habits Scale (family support) (unclear scoring) | End of intervention | 16.0 (9.1); 10 | 14 (9.8); 10 | MD 2.0, 95% CI -6.3 to 10.3 |
|  |  | Social Support for Exercise Habits Scale (friend support) (unclear scoring) | End of intervention | 14.0 (10.0); 10 | 12.5 (3.5); 10 | MD 1.5, 95% CI -5.1 to 8.1 |
|  | Hassett et al.[49] | Goals – Total No. set | End of intervention | 7.4 (1.2); 29 | 7.0 (1.5); 27 | MD 0.4, 95% CI -0.3 to 1.1 |
|  |  |  | End of follow-up | 1.9 (0.8); 28 | 1.7 (0.6); 26 | MD 0.2, 95% CI -0.2 to 0.6 |
|  |  | Goals - percentage of goals achieved (%) | End of intervention | 76.0 (31.0); 29 | 52.0 (31.0); 27 | MD 24.0, 95% CI 7.8 to 40.2 |
|  |  |  | End of follow-up | 68.0 (41.0); 28 | 73.0 (32.0); 26 | MD -5.0, 95% CI -24.5 to 14.5 |
|  |  | Brain Injury Community Rehabilitation Outcome (BICRO) | End of intervention | The BICRO consists of subscales, with a higher score indicating more problems with community reintegration. There were no significant differences between the experimental and control groups at the end of intervention or at follow-up | | |
|  |  |  | End of follow-up |  |  |  |
|  |  | Sydney Psychosocial Reintegration Scale total score (/72 ↑) | End of intervention | 52.9 (14.0); 32 | 56.8 (8.3); 30 | MD -3.9, 95% CI -9.6 to 1.8 |
|  |  |  | End of follow-up | 53.4 (15.8); 32 | 56.9 (13.8); 30 | MD -3.5, 95% CI -10.9 to 3.9 |
|  | Tefertiller et al.[57] | Participation Assessment with Recombined Tools (Objective) (0-5 ↑) | End of intervention | 1.9 (0.1); 30 | 1.0 (0.1); 30 | MD 0.0, 95% CI -0.1 to 0.1 |
|  |  |  | End of follow-up | 1.9 (0.1); 28 | 1.9 (0.1); 30 | MD 0.0, 95% CI -0.1 to 0.1 |
| Physical Activity | Blake et al.[37] | Physical Self-Description Questionnaire (physical activity subscale) (/6 ↑) | End of intervention | 3.5 (3.0); 10 | 1.7 (5.9); 10 | MD 1.8, 95% CI -2.3 to 5.9 |
|  | Kolakowsky-Hayner et al.[36] | Step Count (steps/day) | End of intervention | NR (NR); NR | NR (NR); NR | Not estimable |

NR, Not reported; SD, Standard deviation; MD, Mean difference; CI, Confidence intervals

Where multiple measures have been used in a study for the same, or similar outcome, we have chosen to present data for only the most clinically relevant measure here.

For all scales included in this table, an arrow (i.e. ↑ and ↓) is included to indicate the direction of ‘best’ score for the selected outcome measure i.e. a higher (↑) Berg Balance Score is better

* The SD reported here was taken directly from the Brown et al.[39] publication. The lead author of the paper was contacted to clarify the data but did not respond to our correspondence request.

^ The mobility domain of the Stroke Impairment Scale consists of nine items, scored 1-5, with the highest possible score being 45. It is unclear how this measure has been scored and reported in Esquenazi et al.[46]

⁺ change scores reported
